# Optimization of the structural characteristics of CaO and its effective stabilization yield high-capacity CO_2_ sorbents

**DOI:** 10.1038/s41467-018-04794-5

**Published:** 2018-06-19

**Authors:** Muhammad Awais Naeem, Andac Armutlulu, Qasim Imtiaz, Felix Donat, Robin Schäublin, Agnieszka Kierzkowska, Christoph R. Müller

**Affiliations:** 1 Department of Mechanical and Process Engineering, Laboratory of Energy Science and Engineering, ETH Zurich, Leonhardstrasse 21, 8092 Zurich, Switzerland; 2 Scientific Center for Optical and Electron Microscopy, ETH Zurich, Auguste-Piccard-Hof 1, 8093 Zurich, Switzerland

## Abstract

Calcium looping, a CO_2_ capture technique, may offer a mid-term if not near-term solution to mitigate climate change, triggered by the yet increasing anthropogenic CO_2_ emissions. A key requirement for the economic operation of calcium looping is the availability of highly effective CaO-based CO_2_ sorbents. Here we report a facile synthesis route that yields hollow, MgO-stabilized, CaO microspheres featuring highly porous multishelled morphologies. As a thermal stabilizer, MgO minimized the sintering-induced decay of the sorbents’ CO_2_ capacity and ensured a stable CO_2_ uptake over multiple operation cycles. Detailed electron microscopy-based analyses confirm a compositional homogeneity which is identified, together with the characteristics of its porous structure, as an essential feature to yield a high-performance sorbent. After 30 cycles of repeated CO_2_ capture and sorbent regeneration, the best performing material requires as little as 11 wt.% MgO for structural stabilization and exceeds the CO_2_ uptake of the limestone-derived reference material by ~500%.

## Abstract (web summary)

The economic operation of a carbon dioxide capture technique of calcium looping necessitates highly effective CaO-based CO_2_ sorbents. Here, the authors report a facile one-pot synthesis approach to yield highly effective, MgO-stabilized, CaO-based CO_2_ sorbents featuring highly porous multishelled morphologies.

## Introduction

CO_2_ capture and storage (CCS) is one of the technologies that have been proposed to mitigate climate change stemming primarily from the ever-increasing anthropogenic CO_2_ emissions^1–6^. At present, the capture of CO_2_ by amine scrubbing is a well-established process and industrially applied for the purification of natural gas; yet, it is associated with high costs^7^ and potentially adverse environmental effects through the formation of harmful side-products such as nitrosamines and nitramines^8,9^.

Viable alternatives to amine scrubbing are solid CO_2_ sorbents such as calcium oxide (CaO)^10–15^. The so-called calcium looping process utilizes CaO as a CO_2_ sorbent, and among its most advantageous characteristics are (i) the low cost and wide availability of naturally occurring, environmentally benign CaO precursors (e.g., limestone and dolomite), (ii) the potential use of deactivated material in the cement industry, (iii) the high CO_2_ capture capacity of CaO (~0.78 g_CO2_/g_CaO_), and (iv) the fast kinetics of the underlying capture and release reaction^10–12^:

1 $${\mathrm{CaO}}_{(\mathrm{s})} + {\mathrm{CO}}_{2(\mathrm{g})} \, {{\leftrightarrow}} \, {\mathrm{CaCO}}_{3(\mathrm{s})} \quad \left(\Delta {{H}}^{{\circ}}_{{298} {\mathrm{K}}} = {\pm} {178} \, {\mathrm{kJ mol}}^{-1} \right)$$

However, the main drawback associated with limestone-derived CaO is its low cyclic stability owing to the sintering-induced deterioration of the microstructure of as-derived CaO. Indeed, the Tammann temperature (*T*_T_) of CaCO_3_ (~530 °C), a measure for the onset of sintering, is well below the operating temperatures of calcium looping^11^, i.e., 600–700 °C for carbonation (CO_2_ capture step) and 900 °C for calcination (regeneration step).

One of the approaches that have been attempted to reduce the sintering-induced capacity decay of CaO-based CO_2_ sorbents is the incorporation of high-T_T_ stabilizers such as Al_2_O_3_, MgO, TiO_2_, or ZrO_2_ which are typically inactive for CO_2_ capture. Several works have demonstrated that the utilization of such stabilizers can improve appreciably the cyclic CO_2_ uptake of CaO^10–12,16–21^. In general, stabilizers can be grouped into two categories: (i) the ones that form a solid solution with CaO (e.g., Al_2_O_3_ that can yield Ca_12_Al_14_O_33_ or Ca_9_Al_6_O_18_, or ZrO_2_ forming CaZrO_3_), and (ii) the ones that do not form a solid solution under relevant operating conditions (e.g., MgO or Y_2_O_3_). Although Al_2_O_3_ has received arguably the highest attention so far, the latter group of stabilizers is potentially more attractive in terms of preserving the intrinsically high CO_2_ uptake of CaO, as active CaO is not “consumed” through the formation of a solid solution that is inactive for CO_2_ capture^22^. Furthermore, a recent study focusing on the deactivation mechanism of Ca_3_Al_2_O_6_-stabilized, CaO-based CO_2_ sorbents demonstrated the segregation of Al_2_O_3_ from Cal-Al mixed oxides, leading to a sintering-induced structural collapse of the material after a certain number of cycles^23^. Hence, considering also its high *T*_T_ (i.e., ~1290 °C), MgO is an attractive structural stabilizer. In addition, similar to CaO, MgO is inexpensive and environmentally benign^24^.

The effective incorporation of a stabilizer into a porous CaO structure relies on two key aspects: (i) its homogeneous distribution within the CaO matrix on a nanometer or even atomic level, and (ii) the minimization of its quantity to maintain a high fraction of CaO in the material. However, it is also worth noting that several reports have indicated that the presence of a stabilizer per se is not sufficient to realize sorbents featuring both cyclic stability and a high CO_2_ uptake capacity^12,19,25^. Indeed, also the structure of CaO has to meet certain characteristics, such as being composed of small building blocks typically in the form of nanometer-sized particles/grains^11,20,25^. As in the case of most non-catalytic gas-solid reactions, the carbonation of CaO can occur in two consecutive regimes, i.e., an initial, rapid, kinetically controlled reaction stage that is followed by a relatively sluggish conversion stage. The latter reaction stage is controlled by diffusive limitations, first owing to migration of CO_2_ through the network of pores and later the growth of a CaCO_3_ product layer that has significantly poorer diffusion characteristics for CO_2_ when compared to CaO (*D*_CaO_/*D*_CaCO3_ ≈ 100)^23,26,27^. Considering the relatively short residence times in practical fluidized bed reactors, it is therefore essential that CO_2_ capture is performed largely in the kinetically controlled regime. This can be achieved by ensuring a nano-structured morphology of CaO, minimizing the diffusion lengths of CO_2_ through the freshly formed CaCO_3_ layer. Previous, albeit indirect, measurements have indicated a critical product layer thickness (i.e., the thickness of the product layer when the reaction becomes diffusion limited) of ~50 nm^28,29^. Furthermore, the CO_2_ sorbent should possess a certain level of porosity in order to be able to compensate for the large volumetric changes that occur during cyclic operation, as the molar volume of CaCO_3_ is more than twice as high as that of CaO.

Previous studies aiming at developing stabilized, CaO-based CO_2_ sorbents have utilized various synthesis methods, including wet-mixing^30,31^, precipitation^32^, co-precipitation^33^, sol–gel^34^, flame spray pyrolysis^35^, and re-crystallization^22^. Yet, the majority of these works have fallen short of ensuring a structure that meets all of the essential characteristics outlined above. An additional concern is that some works that have reported an attractive CO_2_ uptake, have tested the materials under unrealistic operating conditions (e.g., regeneration in pure N_2_ at temperatures <900 °C)^36^, or are based on synthesis protocols that rely on environmentally harmful precursors^17,37–39^.

Recently, template-assisted synthesis approaches have been adopted to realize hollow microspheres and nanospheres for a plethora of applications, including catalysis, energy storage, drug delivery etc.^40,41^. These templates, being either hard (e.g., silica, carbon) or soft (e.g., micelles, emulsions), are coated via techniques that typically involve the precipitation of inorganic precursors onto their surface or the layer-by-layer assembly of inorganic particles^42,43^. The final structure, obtained after the selective removal of the template via dissolution, etching or thermal decomposition, is composed of a hollow interior that is contained by an often mesoporous shell^44^. To limit the complexity and to increase the throughput of the synthesis procedure, a favorable approach would involve the simultaneous formation of the template and the precipitation of the compound(s) of interest.

Herein, we report a facile, template-assisted synthesis approach to yield a highly effective, MgO-stabilized, CaO-based CO_2_ sorbent that relies on environmentally benign precursors. The synthesis protocol adopted here features the aforementioned desirable one-pot characteristics. Carbonaceous spheres, formed in situ from a xylose precursor during hydrothermal synthesis, act hereby as a template and encapsulates compounds of Ca and Mg that are precipitated simultaneously via the hydrolysis of urea. Thermal removal of the template yields hollow microspheres with highly porous shells. The resultant structures contain all of the key features that are essential to yield a CaO-based sorbent with a high and cyclically stable CO_2_ uptake. After 30 cycles of calcination and carbonation under “harsh” operating conditions, the CO_2_ uptake of the materials synthesized is 0.50 g_CO2_/g_sorbent_ (equivalent to a capacity retention of 83%), exceeding the capacity of the benchmark limestone by almost 500%.

## Results

### Synthesis of the CO_2_ sorbents

**Fig. 1:**
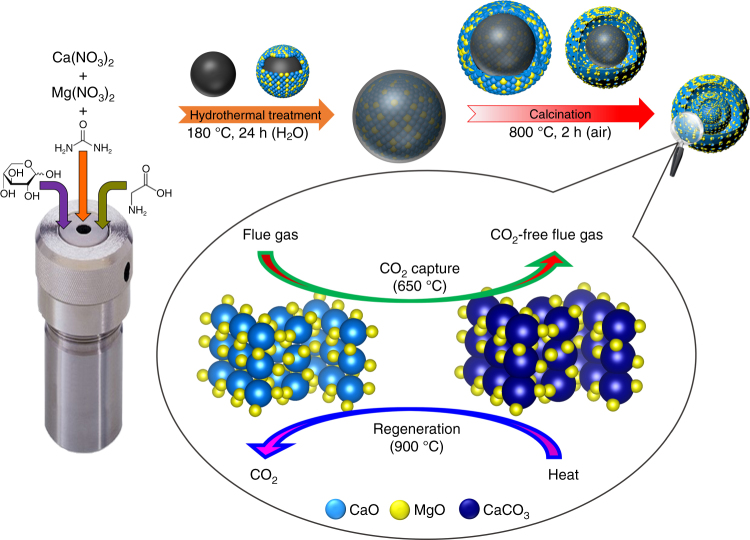
Synthesis protocol. Schematic illustration of the hydrothermal approach developed here to synthesize template-assisted, MgO-stabilized, CaO-based CO_2_ sorbents

As illustrated schematically in Fig. 1, the hydrothermal treatment of an aqueous solution of xylose, urea, and glycine together with Ca and Mg precursors at 180 °C for 24 h yielded micron-sized carbonaceous spheres that encapsulate homogeneously Ca^2+^ and Mg^2+^ species. The simultaneous hydrolysis of urea led to a gradual rise in the pH resulting in the precipitation of CaCO_3_ and MgCO_3_^45–47^. EDX analysis of FIB cross-sections of the as-synthesized material (Supplementary Fig. 1) confirms the homogeneous distribution of Ca and Mg within the carbonaceous template, a rather surprising observation. The presence of glycine in the precursor solution yields complexes of Ca^2+^ and Mg^2+^ which improves the homogeneity of the precipitated CaCO_3_ and MgCO_3_ structures^48^. Xylose was preferred over alternative biomass precursors (e.g., glucose or sucrose), since hydrothermally treated pentoses (e.g., xylose) were shown to yield highly dispersed carbonaceous spheres, whereas the hydrothermal treatment of hexoses (e.g., glucose) resulted typically in an interconnected network of carbonaceous spheres^49^. Calcination of the materials in air at 800 °C resulted in the complete removal of the carbonaceous template and the formation of highly porous, multishelled microspheres, whereby the shells are composed of CaO and MgO nanoparticles. It is noteworthy that all of the compounds utilized in the synthesis of the sorbents are relatively inexpensive and environmentally benign as opposed to other commonly used template-assisted approaches that involve, e.g., resorcinol/formaldehyde-derived templates^50^.

Here, we synthesized CO_2_ sorbents with four different molar ratios of Ca:Mg (i.e., 100:0, 90:10, 85:15, and 80:20). The ratio of Ca to Mg in the materials was determined by ICP-OES, and it was confirmed that the Ca:Mg ratio of the precursors introduced to the reaction vessel was conserved largely throughout the synthesis (Table 1). According to our ICP-OES data, the sorbents Ca90Mg10, Ca85Mg15, and Ca80Mg20 had a MgO content of 8, 11, and 16 wt.%, respectively. These quantities of stabilizers were significantly lower than what has been used so far in most CaO-based CO_2_ sorbents as reported in the literature^11,51^. To achieve a high CO_2_ uptake capacity (on a weight basis), it is essential to minimize the quantity of the CO_2_-inactive stabilizer in the sorbent.

**Table 1:** Composition of the CO_2_ sorbents synthesized and the crystallite sizes of MgO and CaO as determined by ICP-OES and XRD, respectively

Sorbent	Ca:Mg (molar ratio)^a^	CO_2_-active material content (wt.%)^b^	Crystallite size (nm)^c^	
			CaO (200)	MgO (200)
Ca90Mg10	8.9:1.0	~92	43	22
Ca85Mg15	8.5:1.5	~89	45	21
Ca80Mg20	7.8:2.0	~84	46	24

^a^Determined by ICP-OES

^b^Determined by Rietveld refinement

^c^Calculated by the Scherrer Eq. (2) using the XRD data of freshly calcined samples

## Effect of synthesis method on the performance of the sorbents

### Structural characterization of the sorbents

**Fig. 2:**
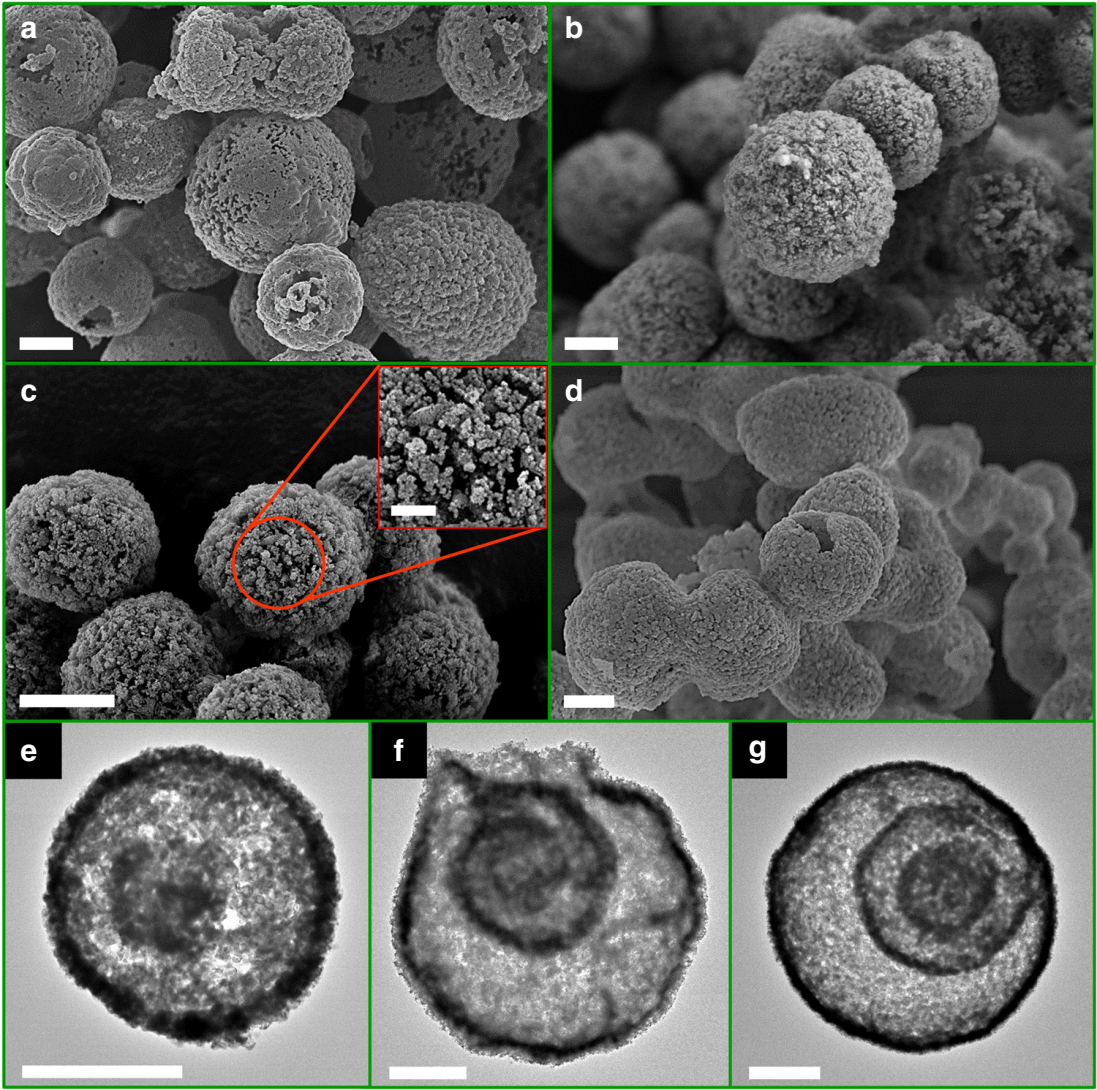
Formation of porous multishelled structures. SEM and TEM images of the hydrothermally synthesized sorbents after thermal removal of the carbonaceous template. **a**, **e** Ca100Mg0. **b**, **f** Ca90Mg10. **c**, **g** Ca85Mg15. **d** Ca80Mg20. Scale bars: 1 μm for **a**–**g**, and 200 nm for inset, respectively

Electron microscopy images of the synthesized materials shown in Fig. 2 reveal that after the thermal removal of the carbonaceous template the desired structure was obtained, i.e., hollow, micrometer-sized spheres composed of highly porous shell(s). Both SEM and TEM images of the samples confirm that variation of the molar ratio of Ca:Mg did not have a noticeable impact on the overall morphology of the materials. The average size of the shell-comprising CaO nanoparticles was determined as ~85 ± 20 nm (Supplementary Fig. 2), i.e., <100 nm, hence fulfilling an essential requirement to avoid (or at least minimize) diffusion limitations during the CO_2_ capture reaction. The average particle size of MgO was measured as 24 ± 5 nm.

The presence of a porous shell is critical as it allows a rapid transport of CO_2_ to and from the material, whereas the central void provides the required volume to accommodate the large volumetric changes the material undergoes during CO_2_ capture and regeneration cycles. Furthermore, the physical separation of the individual nanoparticles is expected to mitigate effectively particle/grain agglomeration^12^. An advantage of the multishelled architecture is an increased packing density (as compared to a single-shelled material), providing, thus, a higher density of CO_2_-active material per volume without significantly compromising the availability of void space^52^.

**Fig. 3:**
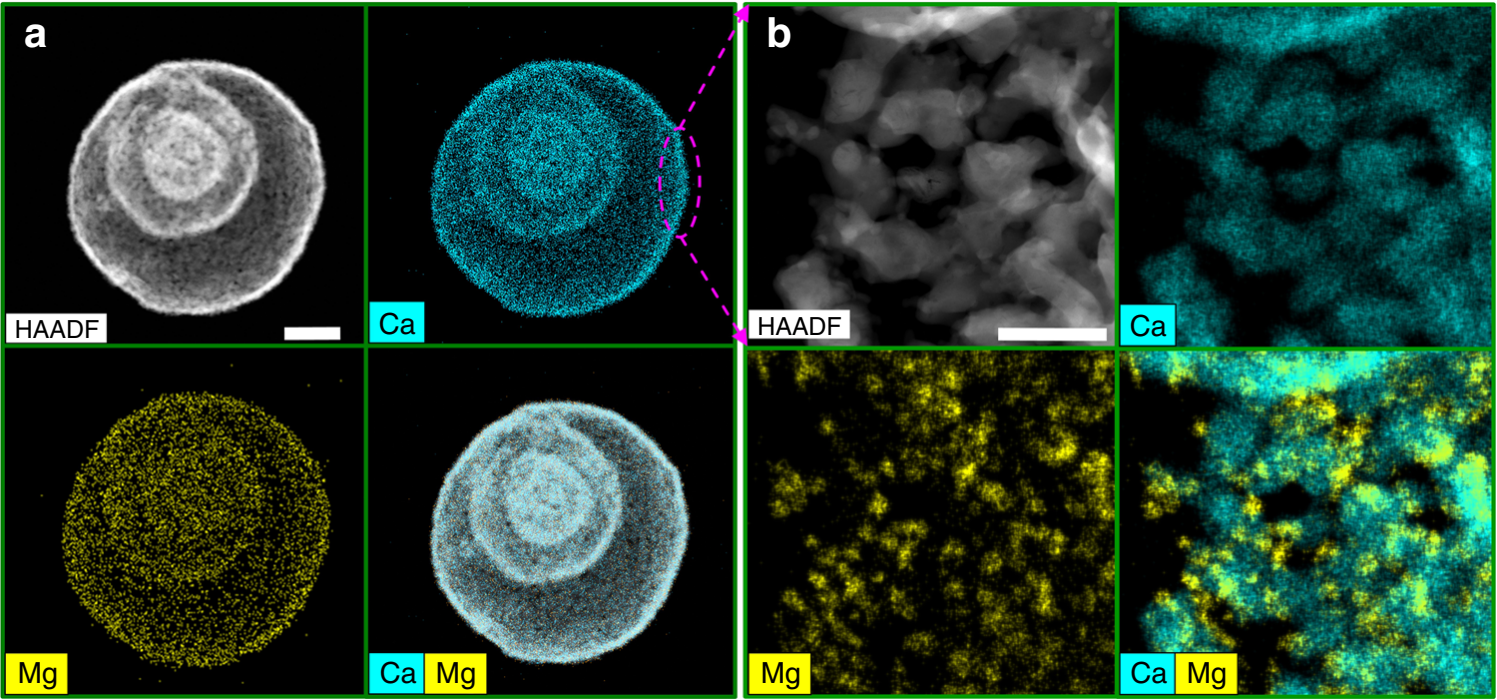
Compositional homogeneity. **a**, **b** Low-magnification and high-magnification HAADF-STEM images of Ca85Mg15 along with EDX elemental mapping showing the distribution of Ca and Mg in the sorbents following the thermal removal of the template, respectively. Scale bar: 1 μm in **a**, and 200 nm in **b**, respectively

To evaluate the compositional homogeneity between CaO and MgO, a feature that is critical to ensure a high thermal stability of the material, HAADF-STEM with EDX analyses were carried out. Figure 3 and Supplementary Fig. 3 show the elemental mappings of Ca and Mg of a multishelled microsphere. Indeed, a homogeneous distribution of MgO within the CaO matrix is observed for both Ca90Mg10 and Ca85Mg15. The high-magnification STEM image complemented by EDX maps (Fig. 3) and the TEM image of the sample (Supplementary Fig. 4) reveal the presence of MgO in the form of uniformly dispersed nanoparticles on the CaO surface. The homogeneous mixing between the active phase and the stabilizer on the nanometer scale is a key requirement for the effective structural stabilization of the sorbent during the cyclic operation, since the functionality of the inert stabilizer (i.e., MgO; *T*_T_ ~1290 °C) is to act as a physical “barrier” between neighboring CaO/CaCO_3_ particles. Such a barrier effectively prevents the agglomeration of the CaO (or CaCO_3_) particles and hence avoids a transition to the diffusion-limited CO_2_ uptake.

**Fig. 4:**
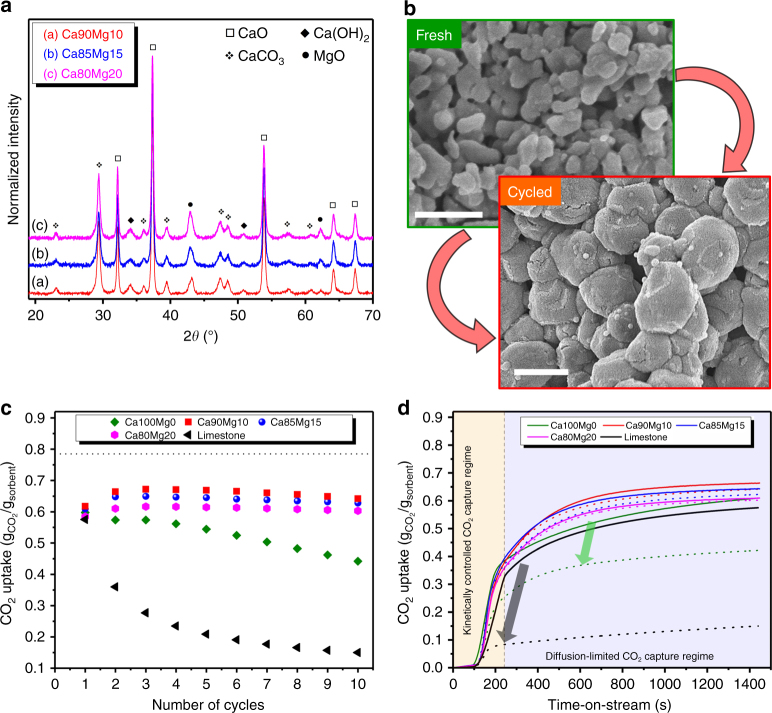
Structure–performance relationship. **a** Powder XRD patterns of the sorbents synthesized after the removal of the carbonaceous template. **b** SEM images of the limestone-derived sorbent in the freshly calcined state and following ten operation cycles (scale bars: 400 nm). **c** CO_2_ uptake of the hydrothermally synthesized CO_2_ sorbents compared to limestone-derived CaO under harsh operating conditions (carbonation at 650 °C, calcination at 900 °C) (dotted line represents the maximum theoretical CO_2_ uptake of Ca100Mg0). **d** Temporally resolved CO_2_ uptake profiles of the hydrothermally synthesized sorbents in the 1st (solid line) and 10th (dashed line) carbonation cycle

XRD measurements, plotted in Fig. 4a, reveal that after the removal of the carbonaceous template, the materials were composed primarily of CaO and MgO. The presence of Ca(OH)_2_ is owing to the highly hygroscopic nature of CaO, whereas CaCO_3_ is believed to form due to the release of CO_2_ during the thermal decomposition of the carbonaceous templates. Rietveld analysis of the sorbents (Supplementary Fig. 5) further confirms that there is no solid solution between CaO and MgO after the thermal removal of the template at 800 °C, suggesting that the homogeneous mixing between the CaO and MgO phases is a purely “physical” phenomenon. Moreover, the crystallite sizes of CaO and MgO, calculated from the XRD data using the Scherrer equation (Eq. 2) (Table 1), indicate that the introduction of MgO does not have a notable impact on the crystallite size of CaO.

### Assessment of the CO_2_ capture performance of the sorbents

The cyclic CO_2_ capture performance of the sorbents was evaluated in a TGA and compared to the benchmark sorbent, i.e., CaO derived from limestone. In order to comply with the practically relevant operation conditions, calcination was performed at 900 °C in a CO_2_ atmosphere, and a rapid temperature ramp of 50 °C min^−1^ was applied between the carbonation and calcination temperatures. For an initial assessment, ten cycles were performed. Already from these initial tests, it is clear that all of the hydrothermally synthesized CO_2_ sorbents show a better performance than the benchmark limestone that underwent significant sintering during cyclic testing (Fig. 4b). In particular, the MgO-containing sorbents exhibit a stable CO_2_ uptake, whereas CaO synthesized in the absence of a stabilizer (i.e., Ca100Mg0) reveals a characteristic decay in the CO_2_ uptake with cycle number (Fig. 4c). This observation is a clear indication of the structural stabilizing effect of MgO. The fact that hydrothermally synthesized CaO without any stabilizer (Ca100Mg0), i.e., a material that initially features structures with favorable CO_2_ capture characteristics, significantly outperforms the limestone-derived benchmark supports the underlying hypothesis that the particular structure of CaO, i.e., being composed of primary particles of <100 nm in diameter and containing sufficient void space to accommodate the volumetric expansion of the material, will yield high CO_2_ uptakes.

The influence of MgO on the performance of the sorbents was examined further by comparing the temporally resolved carbonation profiles of the different CO_2_ sorbents (Fig. 4d). Indeed, all of the MgO-stabilized sorbents maintained their characteristic of capturing a large fraction of CO_2_ in the kinetically controlled regime, in which surface reactions largely prevail (the overlapping CO_2_ uptake curves in the early stages of the carbonation reaction indicate that the reaction in the TGA is dominated by mass transport from the bulk reactive gas to the outer surface of the sorbents). Importantly, the quantity of CO_2_ captured by the MgO-stabilized sorbents in the initial reaction stage was maintained throughout the ten carbonation/calcination cycles (~0.35 g_CO2_/g_sorbent_), whereas unstabilized CaO (Ca100Mg0) experienced an earlier transition to the diffusion-controlled CO_2_ capture regime, decreasing the overall CO_2_ uptake with increasing cycle number (from ~0.34 to 0.22 g_CO2_/g_sorbent_). In Fig. 4d it can also be seen that the transition to the diffusion-controlled reaction stage occurred more gradually for MgO-stabilized sorbents, indicating that a larger pore volume was accessible in these materials. In contrast, the transition from the kinetically controlled to the diffusion-controlled carbonation stage was relatively sharp for limestone and Ca100Mg0, most likely owing to the blockage of narrow pores such that product layer diffusion became rate-limiting at much lower conversions when compared to MgO-stabilized sorbents^53^.

It has been reported that the diffusion of CO_2_ is enhanced in the presence of steam, a typical impurity in a flue gas stream^54,55^. Hence, we performed additional experiments using a mixture of N_2_, CO_2_ and steam (1.5–2 vol.%) during carbonation to assess the effect of steam on the new CO_2_ sorbents (Supplementary Fig. 6). The presence of steam enhanced the apparent rate of CO_2_ uptake significantly for both Ca85Mg15 and Ca80Mg20 and prevented an early transition to the diffusion-limited reaction stage. The total CO_2_ uptake at the end of the 2nd carbonation reaction of Ca80Mg20 increased under wet conditions (i.e., 0.61 vs. 0.64 g_CO2_/g_sorbent_ for dry vs. wet conditions, respectively), confirming the positive effect of steam on the performance of the sorbents.

### Structure–performance relationship of the sorbents tested

Textural parameters, such as the specific surface area and pore volume, were determined by N_2_ physisorption experiments in order to shed light on the structure–performance relationship of the materials (Supplementary Fig. 7 and Supplementary Table 1). In agreement with its cyclic performance, pure CaO obtained from freshly calcined limestone experiences a substantial drop in both its surface area (i.e., from 15 to 2 m^2^ g^−1^) and pore volume (i.e., from 0.168 to 0.009 cm^3^ g^−1^) after ten cycles of carbonation and calcination. When synthesized hydrothermally, pure CaO (i.e., Ca100Mg0) exhibited a higher pore volume after cyclic testing (i.e., 0.043 cm^3^ g^−1^), indicating that the porous multishelled morphology provided some level of thermal stability to the sorbent (Fig. 4c). Ca85Mg15, on the other hand, featured an even higher surface area and pore volume of 7 m^2^ g^−1^ and 0.084 cm^3^/g (i.e., twice as high as Ca100Mg0 and nearly a tenfold increase compared to limestone), respectively, following ten operation cycles. Hence, it can be concluded that the presence of MgO plays a vital role in maintaining the favorable textural properties of the material. When the hydrothermal synthesis was performed in the absence of a carbonaceous template while keeping the Ca:Mg ratio constant at 85:15, the sorbent experienced decays of 93 and 83% in its surface area and pore volume, respectively, during cyclic operation. Similarly, natural dolomite, which served as another reference material, lost more than 87% of its initial pore volume despite its high MgO content of more than 30 wt.%, suggesting that the presence of MgO per se does not suffice to preserve the initially high surface area and pore volume of the sorbent. In light of these observations, it can be concluded that both the presence of MgO and the porous, multishelled morphology of the sorbent are essential characteristics that are required simultaneously to enhance the CO_2_ uptake performance of the material.

The stabilizing effect of MgO was revealed further by electron microscopy. SEM images given in Supplementary Fig. 8 confirm that the MgO-stabilized CO_2_ sorbents undergo only minimal changes in their morphology. On the other hand, pure CaO microspheres “deform” and agglomerate from the first cycle onwards, leading in turn to a decrease in their CO_2_ uptake. It is also noteworthy that the shells of the MgO-stabilized CO_2_ sorbents possess an appreciable degree of porosity even in their carbonated state (Supplementary Fig. 8). This is in contrast to pure CaO microspheres, confirming further the favorable effect of the incorporation of high-*T*_T_ MgO to mitigate sintering and agglomeration of CaCO_3_ during cyclic operation.

Performing ten repeated cycles of calcination and carbonation (Fig. 4c) did not suffice to capture in detail the minimum quantity of MgO required to ensure a stable cyclic behavior of the material. Therefore, MgO-stabilized sorbents were exposed to a higher number of CO_2_ capture and regeneration cycles (Supplementary Fig. 9a). Capacity decays of 36.1, 17, and 17.1% were recorded for, respectively, Ca90Mg10, Ca85Mg15, and Ca80Mg20, outperforming limestone-derived CaO even more significantly (limestone lost more than 80% of its initial CO_2_ uptake capacity after 30 cycles). The almost identical values of capacity retention for Ca85Mg15 and Ca80Mg20 indicate that increasing the MgO content beyond 15 mol% did not improve further the cyclic stability of the material (the addition of increasing quantities of MgO is ultimately a compromise between an increased structural stability of the material and a reduced quantity of CO_2_ capture-active CaO). When the long-term performance of the sorbents was assessed under isothermal conditions at 750 °C (Supplementary Fig. 9b), MgO-stabilized sorbents featured a stable CO_2_ uptake with a negligible capacity decay independent of their MgO content, whereas limestone-sorbent still lost more than half of its initial capacity even under such “mild” operating conditions.

**Fig. 5:**
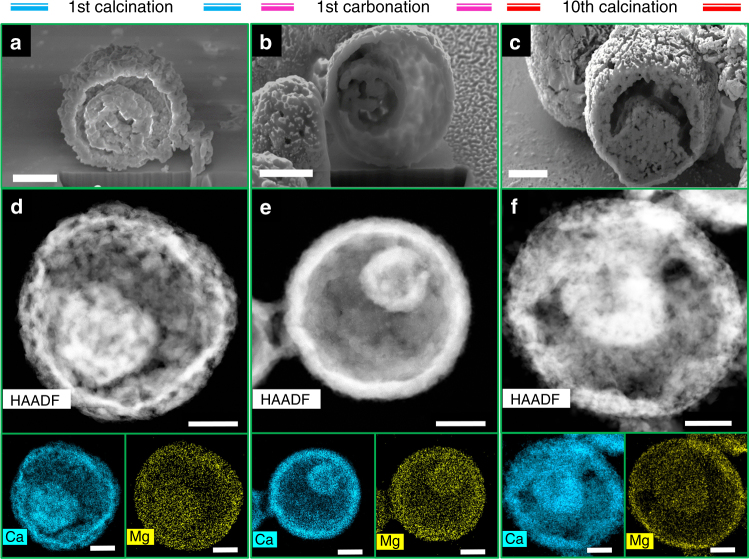
Morphological changes during cyclic operation. **a**–**c** SEM images of FIB cross-sections. **d**–**f** HAADF-STEM images with EDX maps of Ca and Mg for Ca85Mg15 at different stages of CO_2_ capture/regeneration cycles. Scale bars: 1 μm for **a**–**c** and 700 nm for **d**–**f**, respectively

The morphology of the best performing sorbent (i.e., Ca85Mg15) was probed by both FIB-SEM and STEM-EDX at different stages of the cyclic CO_2_ capture and regeneration process (Fig. 5). SEM images of FIB cross-sections show the presence of a multishelled morphology of the as-synthesized CO_2_ sorbents after the removal of the carbonaceous template. In addition to SEM images (Fig. 5a–c), both HAADF-STEM (Fig. 5d–f) and TEM (Supplementary Fig. 10) analyses confirm that the MgO-stabilized sorbents retain their favorable morphology, i.e., hollow spheres with highly porous shells, to a large extent during cyclic operation. This translates in turn to a cyclically stable CO_2_ uptake. Furthermore, EDX maps of Ca and Mg (Fig. 5d–f) indicate that the homogeneous mixing between CaO and MgO at the nanometer scale persists not only in the carbonated state of the sorbent but also in the calcined state after ten operation cycles.

**Fig. 6:**
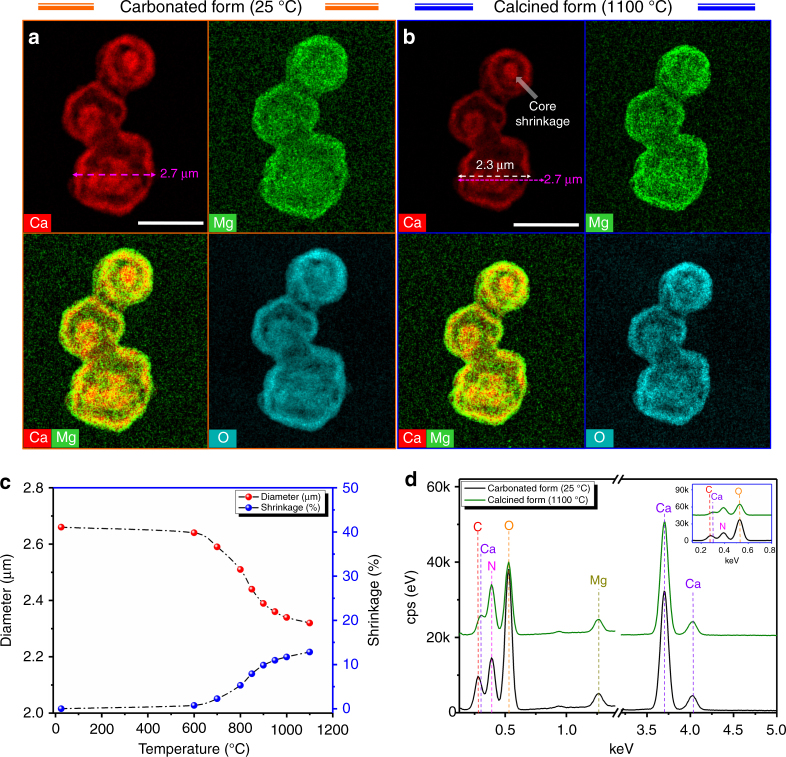
Thermal stability of the multishelled structure. **a** STEM/EDX maps of Ca, Mg, and O in Ca85Mg15 acquired in carbonated form at room temperature. **b** STEM/EDX maps of Ca, Mg, and O in Ca85Mg15 acquired in calcined form at 1100 °C. **c** Change of the size of a microsphere as a function of temperature. **d** EDX spectra of Ca85Mg15 before and after calcination. Scale bars: 2 µm

To capture the structural evolution of the hydrothermally synthesized sorbents during regeneration, in situ TEM was performed on Ca85Mg15 (carbonated state) by heating it up from room temperature to 1100 °C. As shown by the EDX maps of Ca, Mg, and O, provided in Fig. 6a, b, not only the multishelled structure, but also the compositional homogeneity of the sorbent was maintained, even at very high temperatures. It is also noteworthy that although the molar volume of CaCO_3_ is twice as high as that of CaO, the shrinkage in the diameter of the microsphere after being exposed to 1100 °C was determined to be <15% (Fig. 6c). Considering that the peak associated with carbon disappeared and the oxygen count dropped substantially in the EDX spectra at 1100 °C (Fig. 6d), the limited shrinkage cannot be related to an incomplete conversion of CaCO_3_ to CaO, but rather to the favorable morphology of the sorbent, which provides sufficient void to accompany substantial volumetric changes during cyclic operations.

As demonstrated by the electron microscopy studies, the favorable structural features of the hydrothermally synthesized sorbents are preserved to a large extent over several CO_2_ capture-regeneration cycles. The structural stability and compositional homogeneity of the material are believed to be the primary reasons for the cyclically stable performance of the CO_2_ sorbents. To confirm this hypothesis and to probe in more detail the advantages of the template-assisted synthesis approach with regards to the morphology and CO_2_ uptake of the material, alternative single-pot synthesis approaches were tested. These approaches that do not involve a template, include mechanical mixing or hydrothermal treatment in the absence of a carbon precursor. The ratio Ca:Mg was fixed to 85:15. In addition, calcined dolomite (a natural mineral with a nearly equimolar ratio of MgCO_3_ and CaCO_3_) served as a further reference material.

**Fig. 7:**
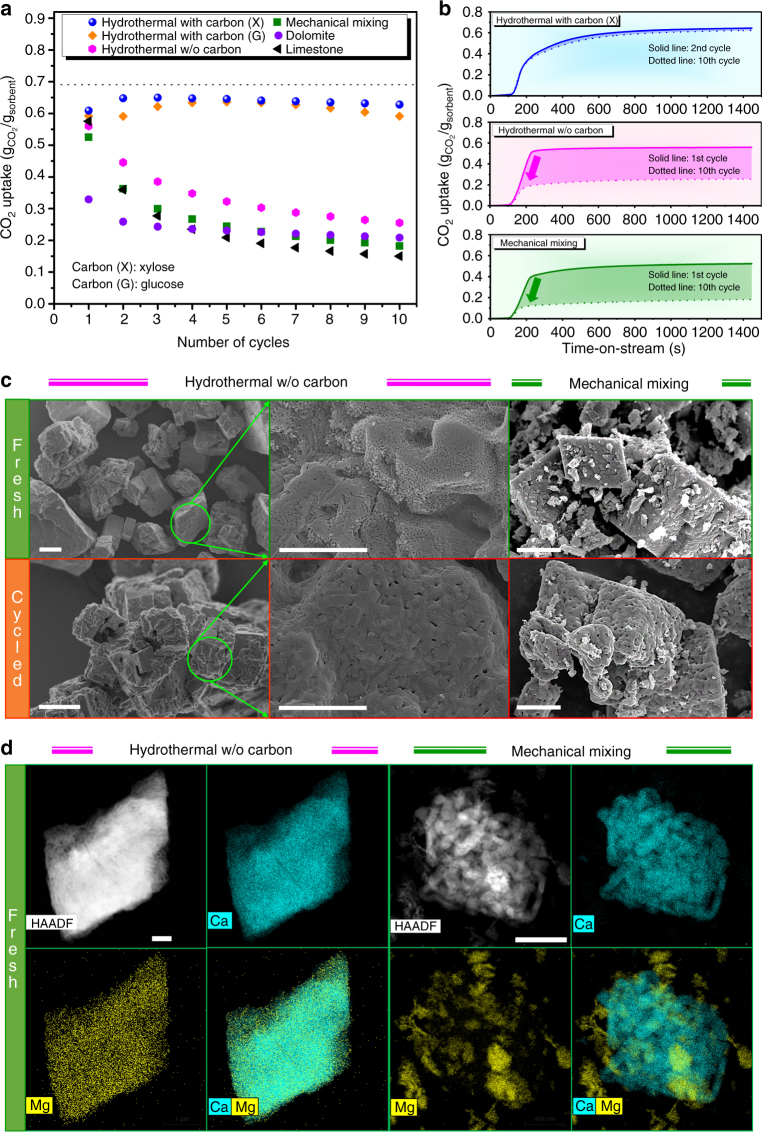
Effect of the synthesis approach on performance and structure. **a** CO_2_ uptake over ten cycles of calcination and carbonation of CO_2_ sorbents that contain a molar ratio of Ca:Mg = 85:15 synthesized by two different approaches: hydrothermal versus mechanical mixing (dotted line represents the maximum theoretical CO_2_ uptake of Ca85Mg15). **b** Temporally resolved carbonation profiles of the sorbents with the highest and lowest CO_2_ uptakes. **c**, **d** SEM images of the as-synthesized and cycled materials, and HAADF-STEM image and EDX mapping of the as-synthesized materials prepared by a hydrothermal route in the absence of a carbon precursor and by mechanical mixing of CaCO_3_ and MgCO_3_. Scale bars in **c**: 20 μm for the 1st column and 2 μm for the 2nd and 3rd columns, respectively. Scale bars: 500 nm in **d**

The cyclic CO_2_ uptake performance of the additional sorbents synthesized is plotted in Fig. 7a, b. The sorbent prepared via the mechanical mixing of CaCO_3_ and MgCO_3_ exhibits a very similar trend as limestone-derived CaO, both in terms of CO_2_ uptake and cyclic stability. Hence, the “simple” presence of a stabilizer (i.e., MgO) is not sufficient to yield a material with a high and cyclically stable CO_2_ uptake. Electron microscopy of the sorbent prepared by mechanical mixing (Fig. 7c) reveals a rather ill-defined structure that is composed of comparatively large building blocks (>2 µm), pointing to the necessity of small particle sizes (both for the stabilizer and CaO) and a high degree of porosity to yield a CO_2_ sorbent that is superior to limestone. In addition, HAADF-STEM/EDX (Fig. 7d) along with SEM/EDX (Supplementary Fig. 11) confirms an inhomogeneous distribution of the stabilizer (MgO) within the active material (CaO) in both the as-synthesized and cycled material. This observation is an indication that also a homogeneous mixing between MgO and CaO (at the nanometer level) is critical for a structural stabilization and in turn, favorable CO_2_ uptake characteristics.

The hydrothermally synthesized material without a carbonaceous template exhibits a slight improvement over the CO_2_ uptake of its mechanically mixed counterpart (Fig. 7a, b), albeit revealing a significant capacity decay with cycle number. Interestingly, SEM images show that also this material has a rather ill-defined morphology that is composed of large particles (>2 µm) (Fig. 7c). The CO_2_ uptake of the template-free material is appreciably lower than that of the CO_2_ sorbents that were obtained via a template-assisted hydrothermal route. These observations suggest that, although the hydrothermal synthesis approach enables a compositional homogeneity between MgO and CaO as demonstrated by HAADF-STEM/EDX (Fig. 7d) and SEM/EDX analyses (Supplementary Fig. 12), a homogeneous mixing per se is not sufficient to yield a high and cyclically stable CO_2_ uptake. Similarly, also dolomite contains a relatively homogeneous distribution of MgO nanoparticles within the CaO matrix (Supplementary Fig. 13), which is in line with previously reported studies^56,57^. Although dolomite-derived sorbents surpass the CO_2_ uptake of their limestone-derived counterpart from the 4th cycle onward, the improvement was only marginal and the hydrothermally synthesized sorbents outperformed significantly dolomite (Fig. 7a). The notable capacity decay of dolomite-derived sorbents, despite their high content of MgO, can be explained by the segregation of MgO nanoparticles out of the CaO matrix, thereby losing their stabilizing role. This is visualized very well by electron microscopy (Supplementary Fig. 13).

These findings suggest that the presence of a void (providing the space required for the volume expansion during carbonation and mitigating agglomeration and structural collapse) and limiting the size of the primary CaO particles to diameters <100 nm are features that are critical to obtain CaO-based materials with superior CO_2_ uptake characteristics. Besides the macrostructure of the sorbents synthesized, XRD results reveal that the template also plays an important role in the crystallite size of CaO (Supplementary Fig. 14 and Supplementary Table 2). When synthesized hydrothermally in the presence of a carbonaceous template, the sorbents feature relatively small crystallite sizes of ~45 nm that is largely preserved over ten cycles (minor increase to ~48 nm). On the other hand, sorbents synthesized hydrothermally in the absence of a template had an initial crystallite size of ~73 nm and exceeded 100 nm after cyclic operation. As a final note, when we replaced xylose by other inexpensive and widely available carbon precursors (i.e., glucose), materials with similar morphologies (Supplementary Fig. 15) and comparable CO_2_ uptakes (Fig. 7a) were obtained.

## Discussion

In summary, we report a facile one-pot synthesis approach to yield highly effective, MgO-stabilized, CaO-based CO_2_ sorbents. When mixed homogeneously with CaO on the nanometer level, MgO acts as an inert, sintering retarding structural stabilizer. The presence of a carbonaceous template during synthesis allowed (after its removal) for the formation of a well-defined morphology featuring multishelled structures with a high degree of porosity; a critical requirement for a high CO_2_ uptake. By assessing alternative synthesis approaches, we demonstrate that the compositional homogeneity and the formation of a (multi)shelled morphology, containing excess porosity and being composed of small nanoparticles, are key requirements that have to be met to obtain effective CO_2_ sorbents. Using a template-assisted hydrothermal approach, a MgO content of as little as 15 mol% (i.e., 11 wt.%) is sufficient to obtain a high level of cyclic stability. The best sorbent retained 83% of its initial CO_2_ uptake over 30 CO_2_ capture and regeneration cycles under practically relevant operating conditions, outperforming the limestone-derived benchmark by ~500%.

## Methods

### Synthesis protocol for the sorbents

MgO-stabilized, multishelled CaO-based microspheres were synthesized via a hydrothermal approach. In a typical synthesis, d-(+)-xylose (40 mmol; Sigma Aldrich) and glycine (6.6 mmol; Acros Organics) were dissolved in 15 ml of deionized (DI) water under vigorous stirring for 10 min. Next, calcium and magnesium precursors, i.e., Ca(NO_3_)_2_·4H_2_O and Mg(NO_3_)_2_·6H_2_O (from Acros Organics) were added in appropriate quantities (in total 17 mmol with different molar ratios of Ca:Mg of 90:10, 85:15, and 80:20) to the xylose solution under vigorous stirring. After mixing for 10 min, 3 ml of an aqueous urea solution (2 M) was introduced to the reaction mixture. Following vigorous stirring for 5 min, the solution was transferred to a 45-ml Teflon-lined stainless-steel autoclave. The autoclave was kept in an oven at 180 °C for 24 h. After cooling down to room temperature, a black, powdery material was collected by filtration and washed thoroughly with DI water and ethanol. Drying of the filtrate at 80 °C overnight and subsequent calcination in a muffle furnace at 800 °C for 2 h (5 °C min^−1^ ramp rate) yielded the final CO_2_ sorbent. For comparison, samples with the aforementioned Ca:Mg molar ratios were also prepared by mechanical mixing of CaCO_3_ and MgCO_3_. Following the mechanical mixing of appropriate amounts of CaCO_3_ and MgCO_3_ for 20 min, the samples were calcined in a muffle furnace at 800 °C for 2 h (5 °C min^−1^ ramp rate).

For simplicity, the following nomenclature is used to describe the composition of the CO_2_ sorbents throughout the text: The elemental abbreviations (“Ca” and “Mg” for CaO and MgO, respectively) are followed by a number that quantifies the molar composition of the sorbent. For example, the sorbent Ca85Mg15 contains a molar ratio of Ca:Mg of 85:15.

### Performance characterization

The cyclic CO_2_ uptake of the sorbents synthesized was evaluated in a thermogravimetric analyzer (TGA, Mettler Toledo TGA/DSC 1) at atmospheric pressure. A ramping rate of 50 °C min^−1^ was used for all heating and cooling cycles. The sample (typically ~5 mg) was loaded in a 70-µl alumina crucible and heated to 900 °C under a nitrogen flow (120 ml min^−1^ including the constant purge flow of 25 ml min^−1^ over the microbalance). After calcination (5 min), the sample was cooled down to 650 °C and the cyclic test started with the carbonation reaction (20 vol.% CO_2_ in N_2_ for 20 min). Following carbonation, the sorbent was regenerated by increasing the temperature to 900 °C in a CO_2_ atmosphere. To ensure a complete regeneration of the sorbent, the sample was maintained at 900 °C for 10 min. These calcination and carbonation steps were repeated for the desired number of cycles. Throughout the operation, the weight change of the sorbent was continuously monitored and recorded.

### N_2_ physisorption

N_2_ physisorption experiments were performed in a NOVA 4000e analyzer (Quantachrome Instruments). The adsorption and desorption of N_2_ were determined at −196 °C. Prior to the N_2_ physisorption measurements, the samples were degassed under vacuum at 300 °C for at least 3 h. Brunauer–Emmett–Teller (BET) and Barrett–Joyner–Halenda (BJH) models were used to calculate, respectively, the surface area and the pore-size distribution of the materials.

### X-ray diffraction

Powder XRD (Bruker, AXS D8 Advance) was utilized to investigate the crystallinity and the chemical composition of the materials synthesized. The diffractometer was equipped with a super-speed Lynxeye detector which was operated at 40 mA and 40 kV (2*θ* range of 10–90° with a step size of 0.025° and a step duration of 0.8 s). Rietveld refinement analysis of the XRD patterns was performed using FullProf. The average crystallite sizes (*D*) of CaO and MgO were estimated using the Scherrer Eq. (2):

2 $$D = \frac{{K\lambda }}{{\beta {\rm{cos}}\theta }}$$

where *K* is the shape factor (i.e., 0.9 for a spherical particle), *λ* is the wavelength of the X-ray radiation used (0.15418 nm), *β* is the full width at half maximum intensity (FWHM), and *θ* is the Bragg angle (peak position) of the corresponding reflection.

### ICP-OES

Inductively coupled plasma–optical emission spectroscopy (ICP-OES) was performed on a Varian 720-ES. For calibration, the multi-element standard 5 (Sigma Aldrich) was used. Samples were prepared by treating ~3 mg of the sample with 4 ml of aqua regia (3:1 HCl:HNO_3_). The resulting solutions were diluted with DI water to yield a volume of 25 ml. To avoid any contamination, trace-grade acids (HCl and HNO_3_), and HNO_3_-leached glassware were used.

### Electron microscopy

A high-resolution field-emission SEM (Zeiss ULTRA 55 plus) was utilized to visualize the materials. Prior to imaging, the samples were sputter-coated (BAL-TEC SCD-050) with Au/Pd to improve their conductivity. For the elemental mapping of Ca and Mg, a Leo Gemini 1530 equipped with an EDX detector was used.

Cross-sections of the synthesized microspheres were prepared by focused ion beam (FIB) equipped with a Ga liquid metal ion source and imaged with a high-resolution FE-SEM (Zeiss, FIB-SEM NVision 40). Prior to FIB-SEM analysis, the samples were dispersed and stabilized on a Si wafer.

The microstructure of the materials was probed by TEM (FEI Talos F200X) equipped with a high-brightness field-emission gun, a high-angle annular dark field (HAADF) detector, and a large collection-angle EDX detector. The operation voltage of the instrument was set to 200 kV in both TEM and scanning TEM (STEM) modes. For the ex situ analysis of the samples, the powders were dispersed onto Cu grids coated with lacey carbon.

For the in situ heating experiments, the sample was dispersed onto MEMS chips with SiN_x_ windows (Wildfire D6, DENSsolutions). The sample was heated up from 25 to 1100 °C at a ramp rate of 50 °C min^−1^ and then rapidly cooled down to room temperature.

### Data availability

The data supporting the findings of this work are available from the corresponding author upon reasonable request.

## Electronic supplementary material

Supplementary Information
