# Supplementary material for: Optimization of the structural characteristics of CaO and its effective stabilization yield high-capacity CO2 sorbents
Source: Nat Commun. 2018 Jun 19;9:2408. doi: 10.1038/s41467-018-04794-5 (PMC6008298; doi:10.1038/s41467-018-04794-5)
Supplement: Supplementary file 1 — Supplementary Information [file 41467_2018_4794_MOESM1_ESM.pdf]

SUPPLEMENTARY INFORMATION

Optimization of the structural characteristics of CaO and its effective stabilization yield high-capacity CO_2 sorbents

Muhammad Awais Naeem[a], Andac Armutlulu[a], Qasim Imtiaz[a], Felix Donat[a], Robin Schäublin[b], Agnieszka Kierzkowska[a] and Christoph Müller[a,*]

[a]Laboratory of Energy Science and Engineering, Department of Mechanical and Process Engineering, ETH Zurich, Leonhardstrasse 21, 8092 Zurich, Switzerland

[b]Scientific Center for Optical and Electron Microscopy, ETH Zurich, Auguste-Piccard-Hof 1, 8093 Zurich, Switzerland

*Corresponding author. Tel.: +41 44 632 3440.

E-mail address: muelchri@ethz.ch (Prof. Christoph Müller)

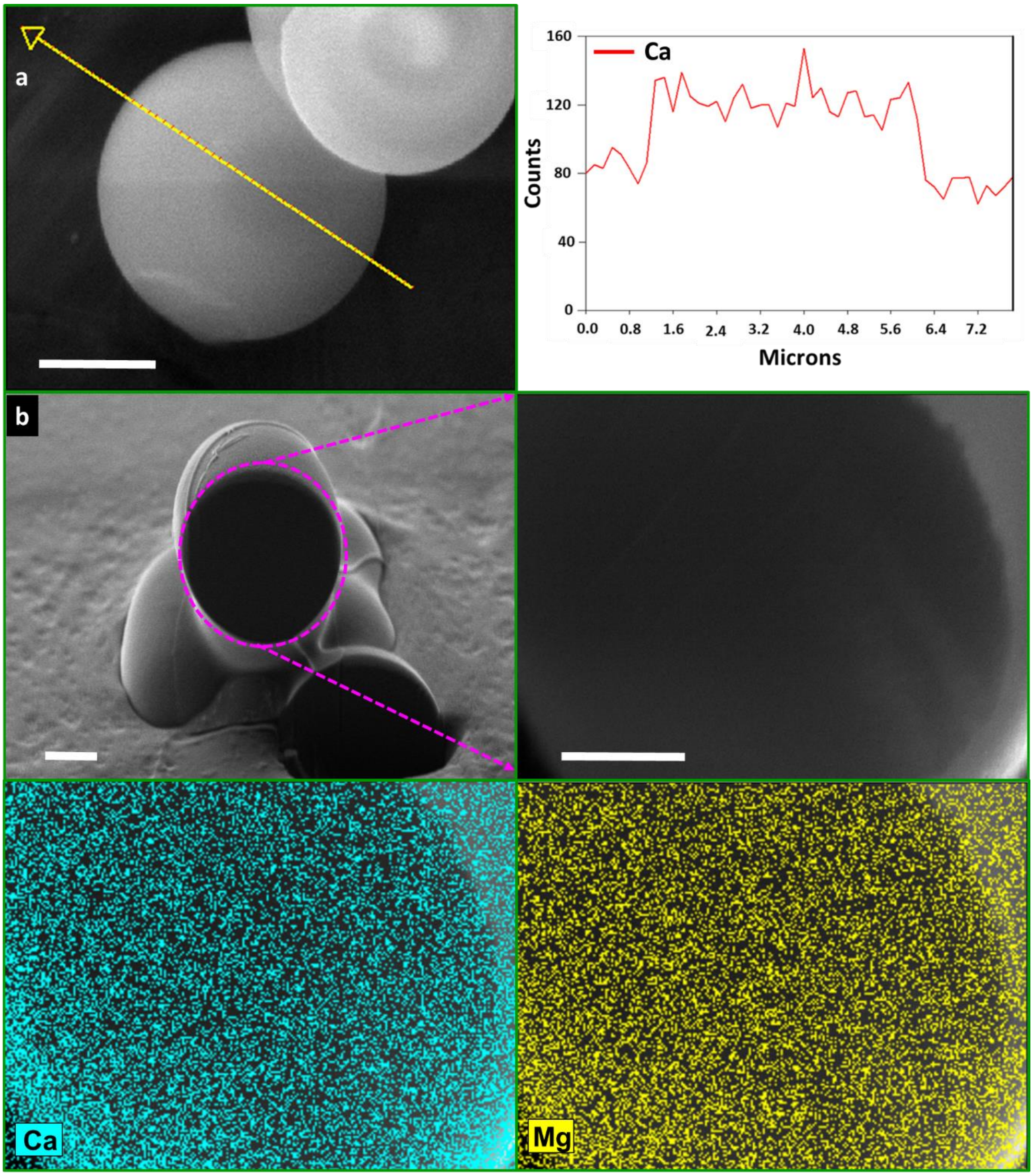

Supplementary Figure 1 Distribution of Ca and Mg within the carbon template. **a** EDX line scan showing the Ca profile across a carbonaceous sphere following hydrothermal synthesis, **b** FIB cross-section of a carbonaceous sphere and EDX analysis thereof showing the homogeneous distribution of Ca and Mg across the carbonaceous sphere. Scale bars: 2 µm for **a**, **b** and 1 µm for the magnified section, respectively.

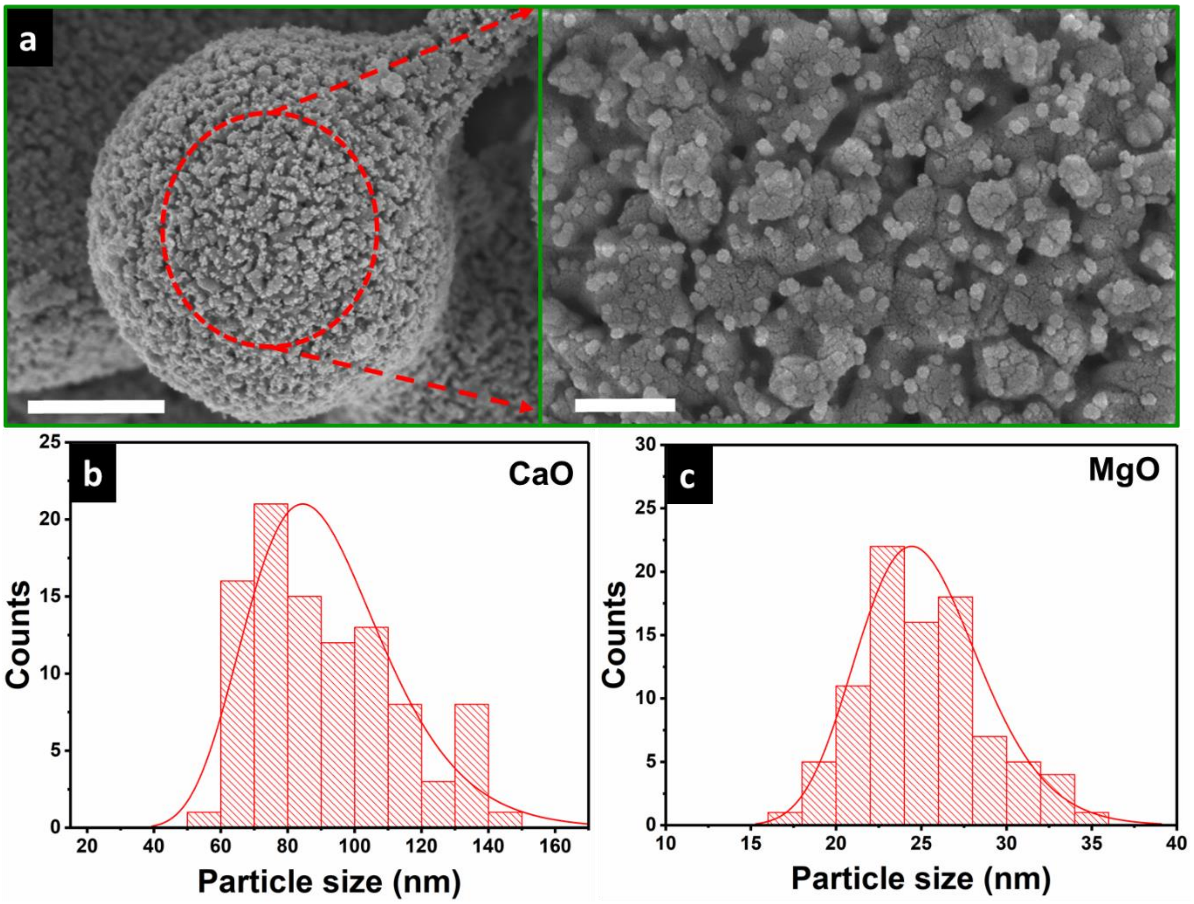

Supplementary Figure 2 Particle size of CaO and MgO in the sorbents. **a** High-resolution SEM images of Ca85Mg15 showing the shell-comprising CaO and MgO nanoparticles, and the size distribution of **b** CaO, and **c** MgO nanoparticles. Scale bars: 1 µm for **a**, and 200 nm for the magnified region, respectively.

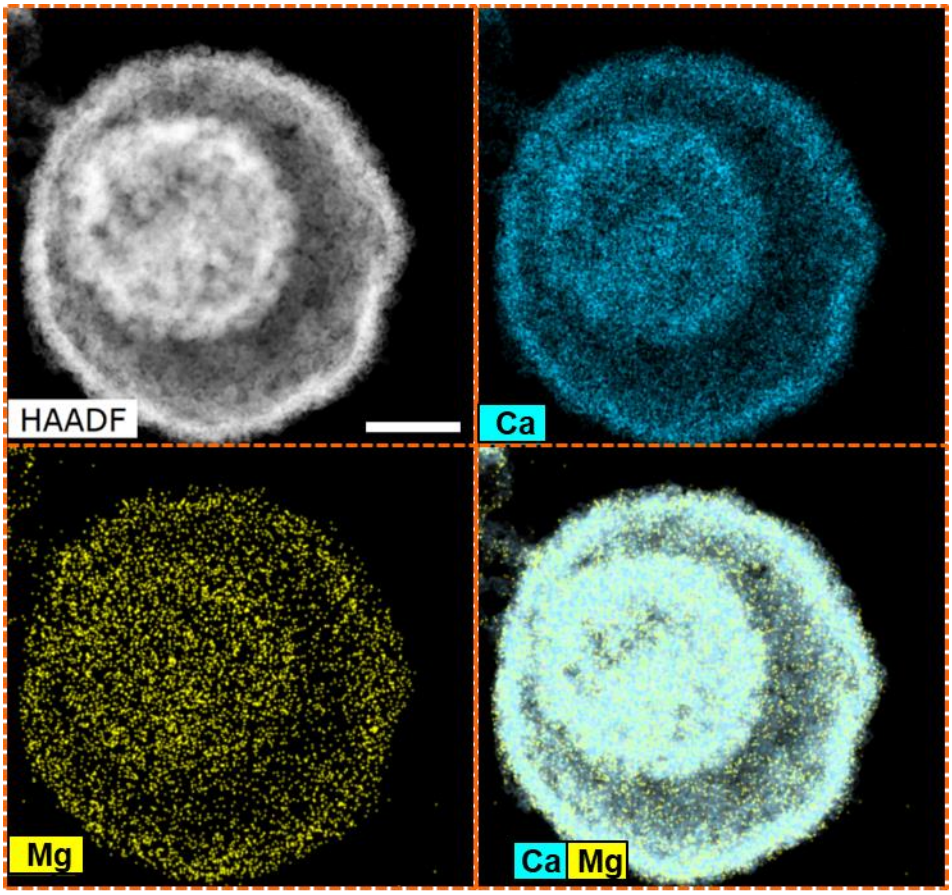

Supplementary Figure 3 Compositional homogeneity in the multishelled structure. HAADF-STEM image of Ca90Mg10 along with EDX elemental mapping showing the distribution of Ca and Mg in the sorbent following the thermal removal of the template. Scale bar, 1 µm.

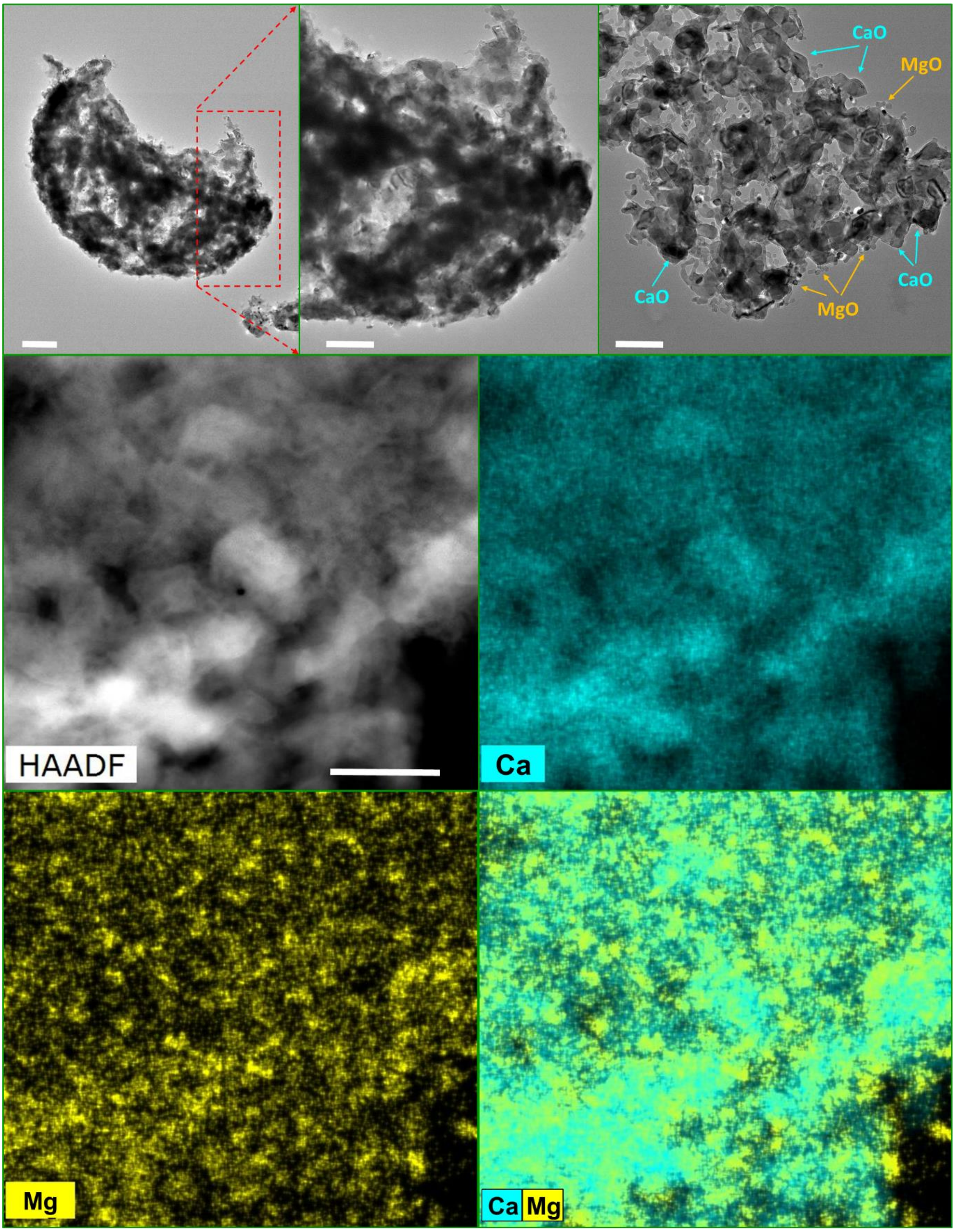

Supplementary Figure 4 Building blocks of the sorbents synthesized. TEM images showing CaO and MgO nanoparticles constituting the microspheres, and STEM along with EDX mappings of Ca and Mg showing the homogeneously distributed MgO nanoparticles within the CaO matrix of Ca85Mg15. Scale bars: 200 nm.

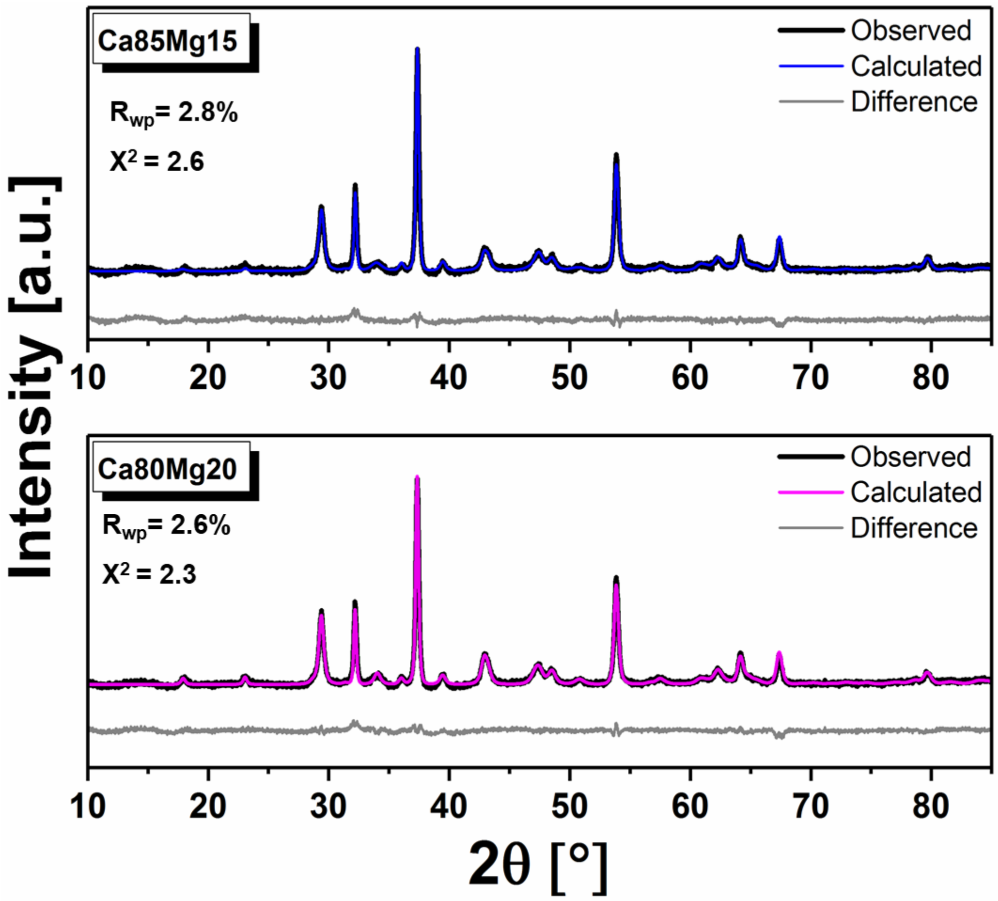

Supplementary Figure 5 Rietveld refinement of Ca85Mg15 and Ca80Mg20.

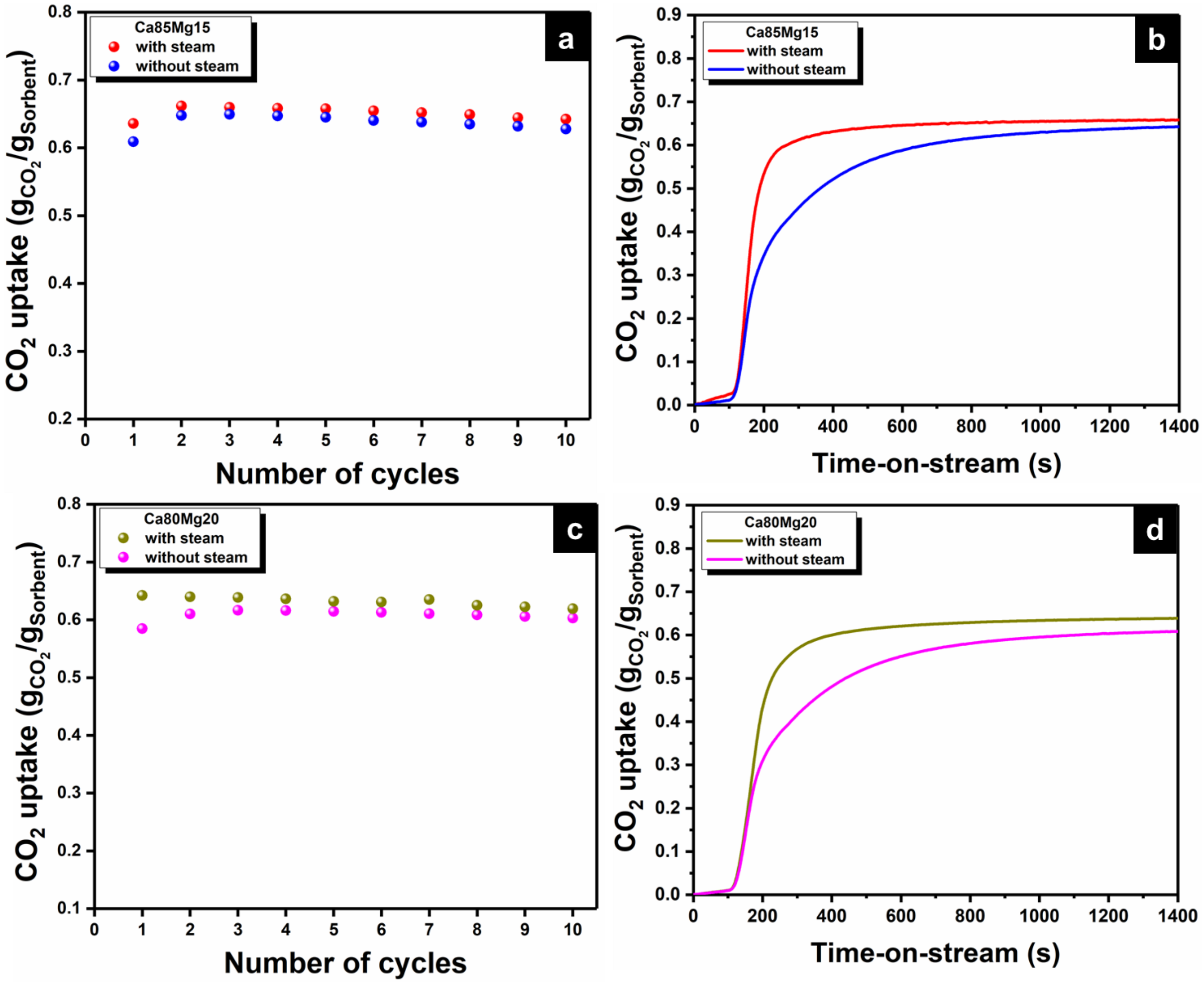

Supplementary Figure 6 Performance of the sorbents in the presence of steam. CO_2 uptake performance of **a** Ca85Mg15 and **c** Ca80Mg20 in the presence and absence of steam over 10 cycles of carbonation and calcination and temporally resolved CO_2 uptake profiles of **b** Ca85Mg15 and **d** Ca80Mg20 during the 2nd carbonation cycle in the presence and absence of steam.

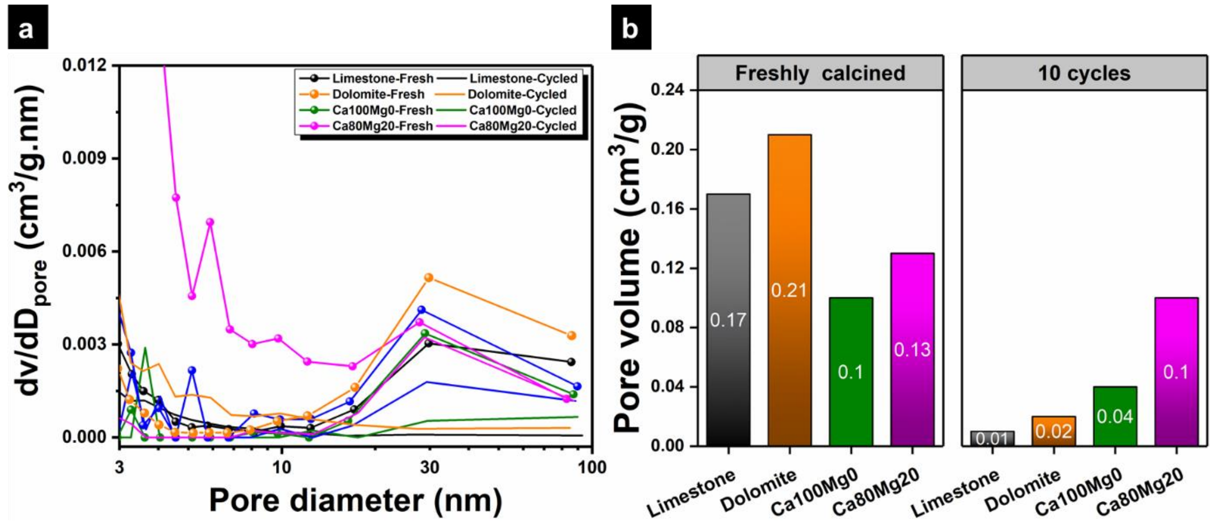

Supplementary Figure 7 N_2 physisorption data of the sorbents tested. **a** Pore size distribution, and **b** pore volume of freshly calcined limestone, dolomite, Ca100Mg0, and Ca80Mg20 before and after 10 cycles of carbonation and calcination.

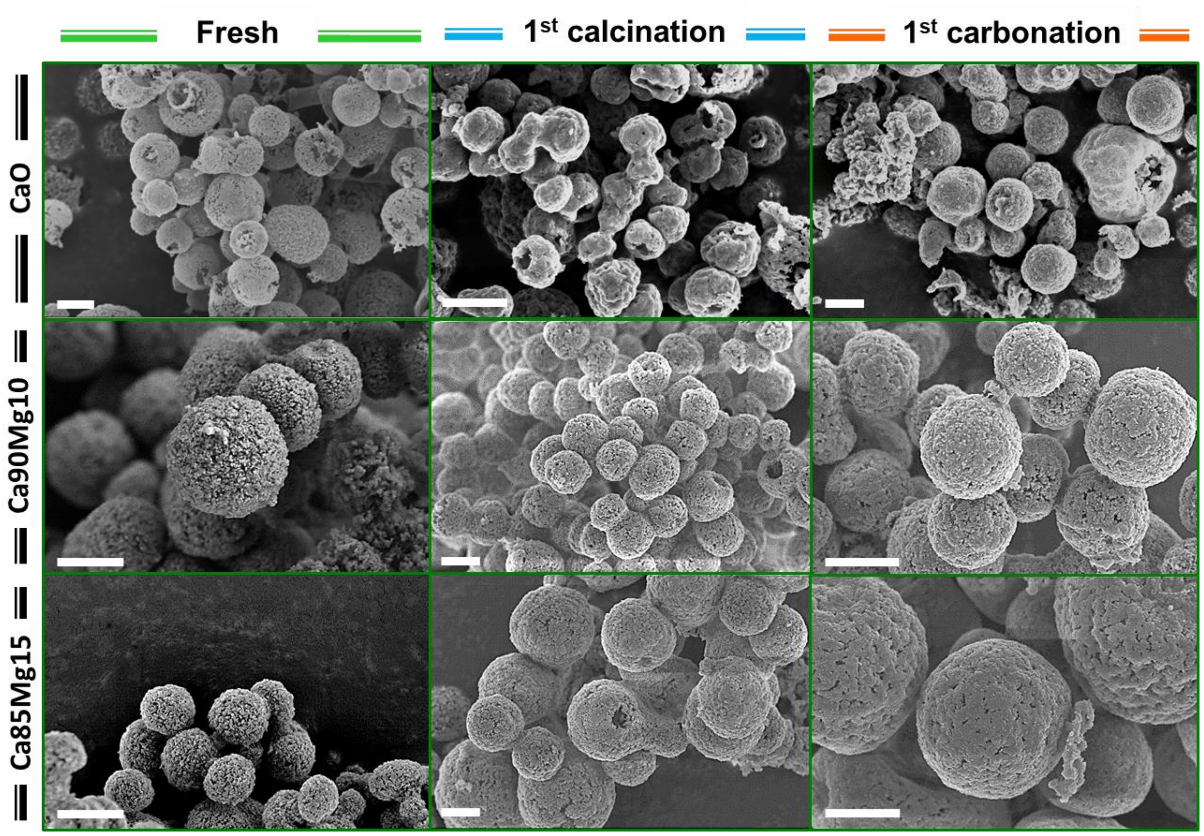

Supplementary Figure 8 Effect of MgO on the structural stability of CaO. SEM images comparing the morphology of CO_2 sorbents synthesized in the absence (CaO) and the presence of MgO (Ca90Mg10 and Ca85Mg15) prior to cyclic tests and after 1st calcination and carbonation cycles. Scale bars: 2 µm

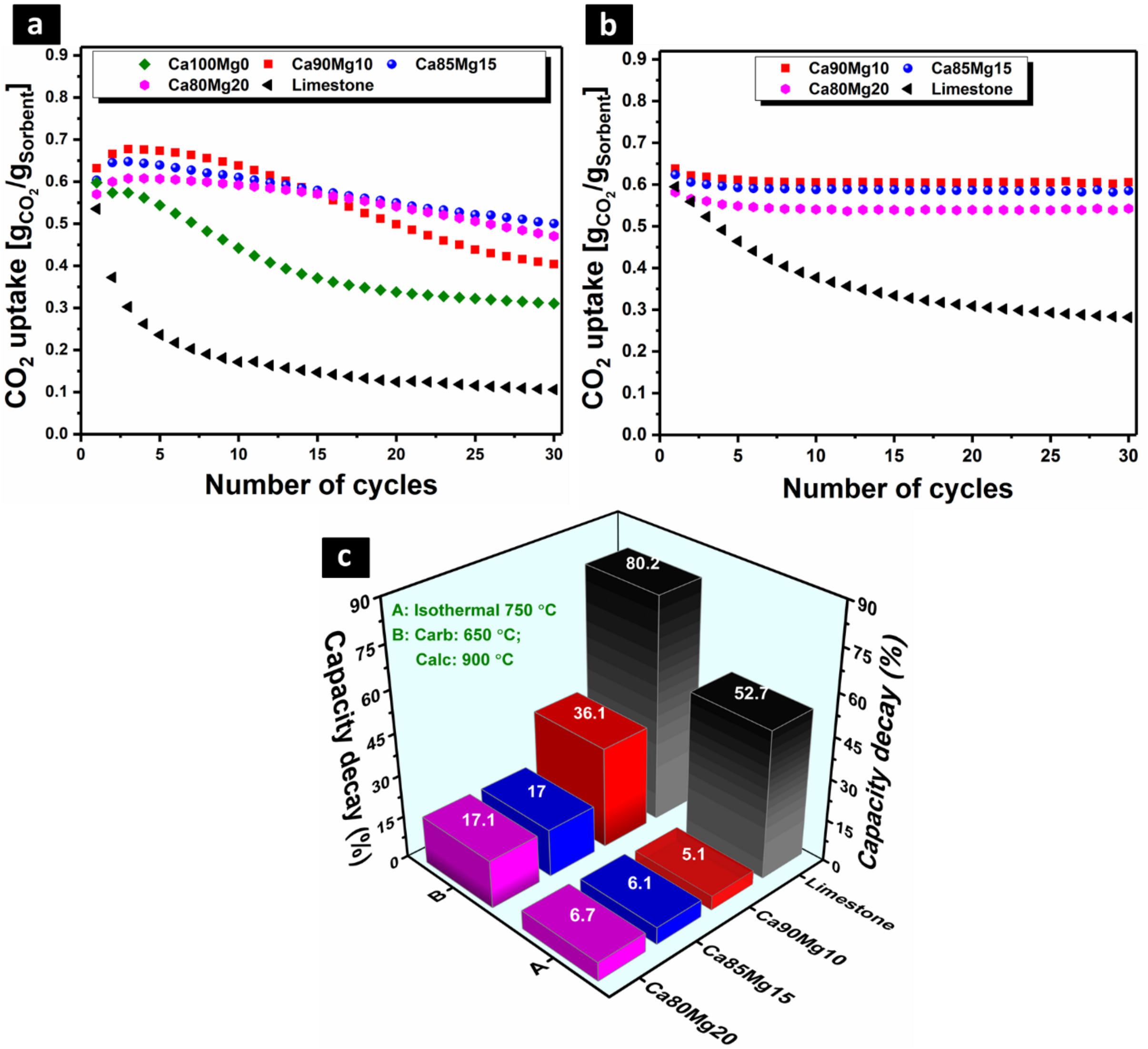

Supplementary Figure 9 Performance under harsh vs mild operating conditions. CO_2 uptake performance of the sorbents synthesized compared to limestone-derived CaO over 30 cycles of carbonation and calcination under **a** realistic (harsh) operating conditions, and **b** isothermal (mild) conditions at 750 °C; **c** capacity decay as a function of sorbent type and operating conditions.

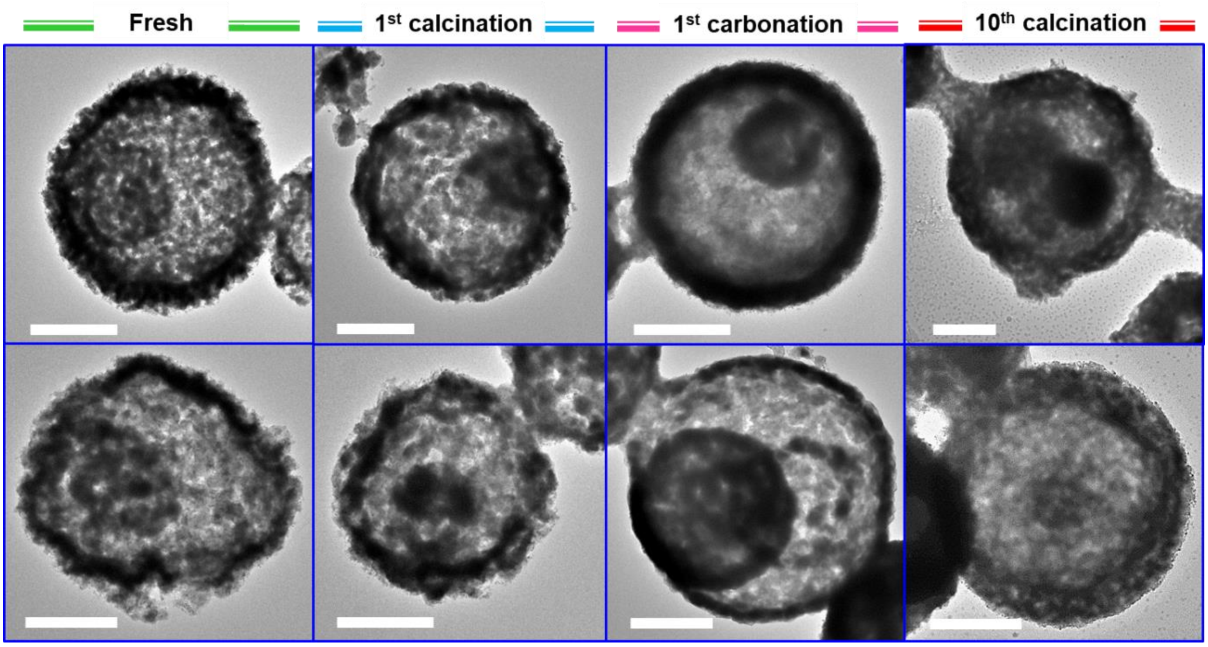

Supplementary Figure 10 Effect of cyclic operation on the morphology of the sorbents. TEM images showing the morphological changes which Ca85Mg15 undergoes under reactive conditions. Scale bars: 1 µm.

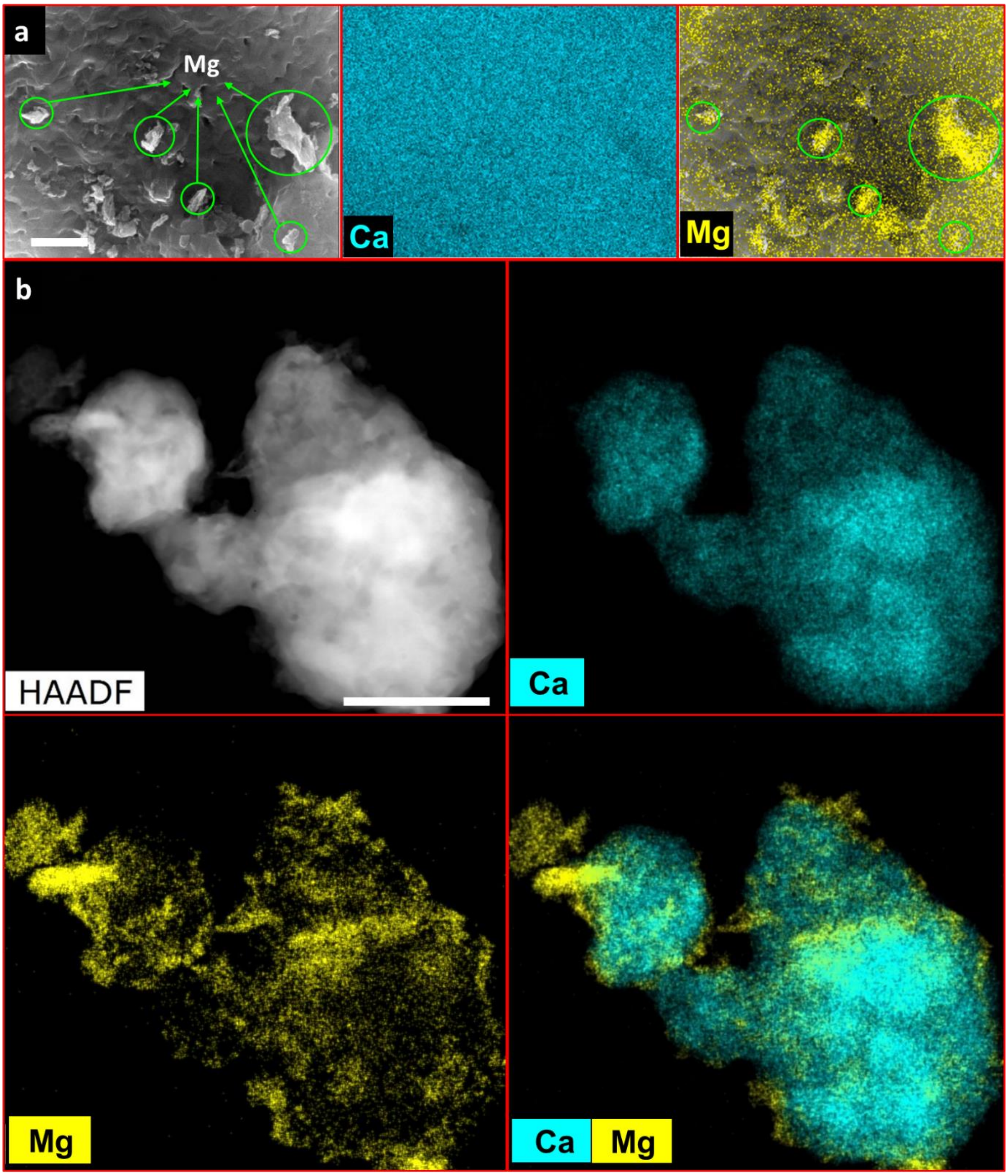

Supplementary Figure 11 Effect of wet mixing on the compositional homogeneity. **a** SEM/EDX and **b** HAADF STEM image with EDX mapping showing the distribution of Ca and Mg in the sample prepared via mechanical mixing after 10 cycles of carbonation and calcination. Scale bars: 1 µm.

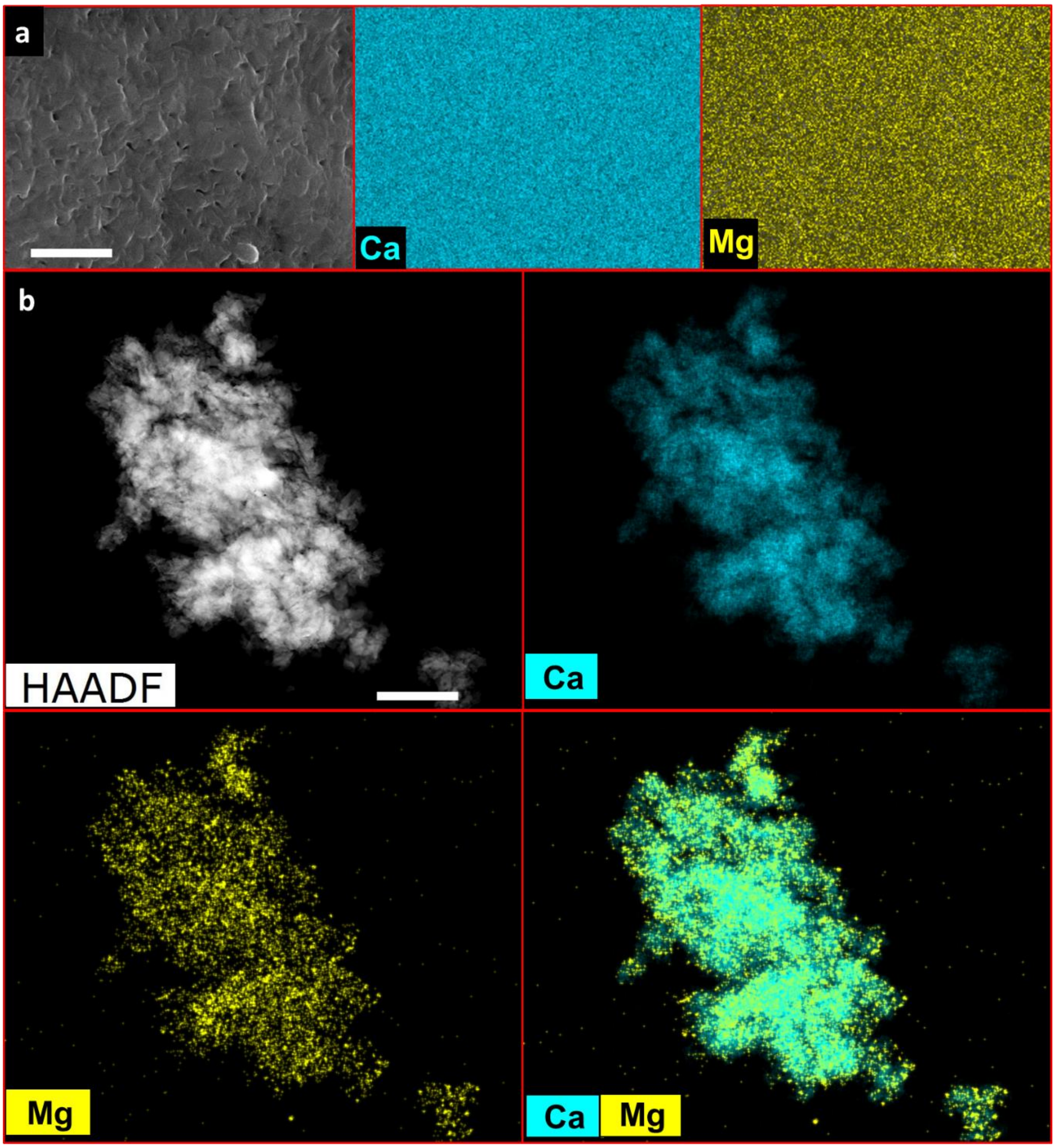

Supplementary Figure 12 Compositional homogeneity in the absence of template. **a** SEM/EDX and **b** HAADF STEM image with EDX mapping showing the distribution of Ca and Mg in the sample prepared via a hydrothermal approach in the absence of a carbon template after 10 cycles of carbonation and calcination. Scale bar: 3 μm in **a**, and 1 μm in **b**, respectively.

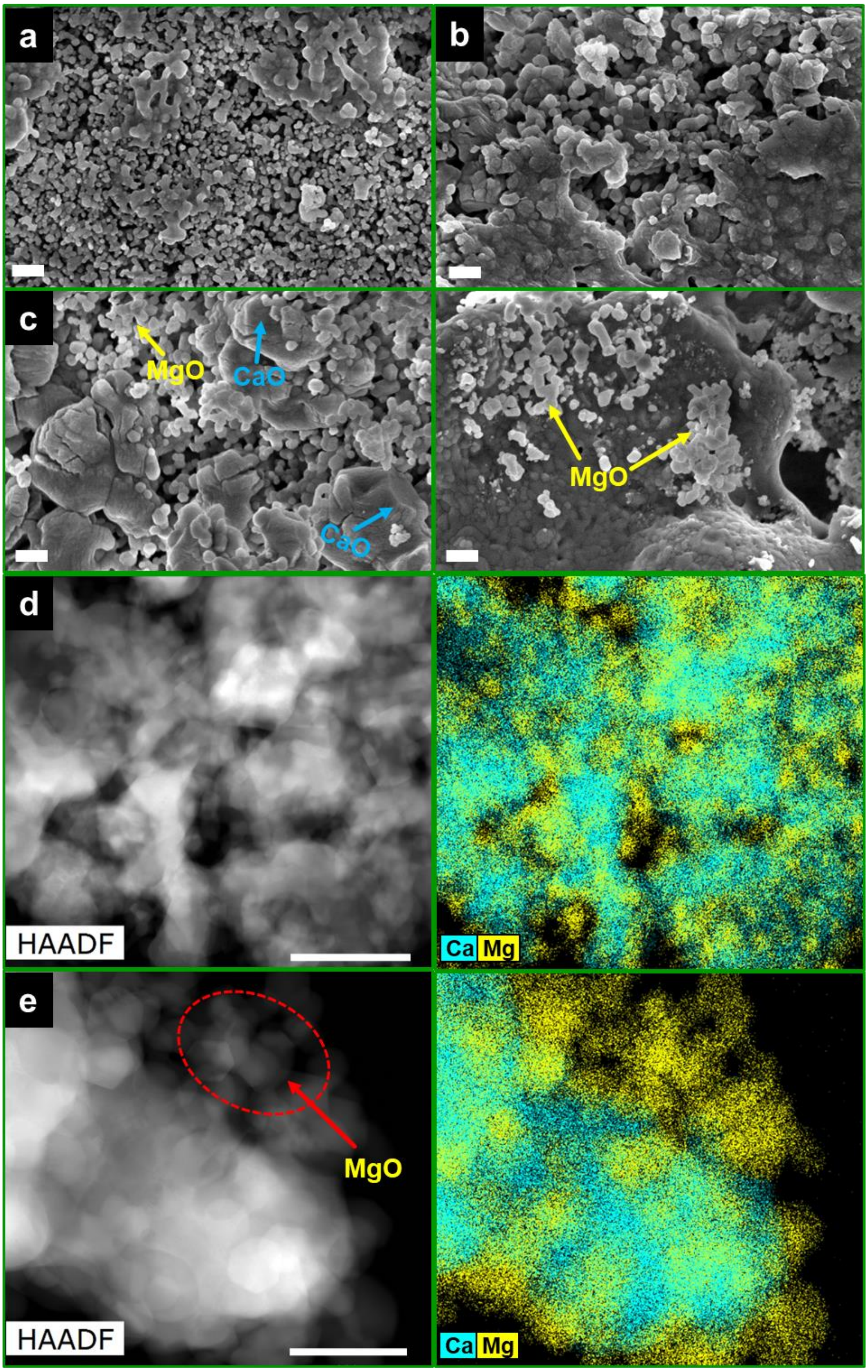

Supplementary Figure 13 Compositional homogeneity of dolomite. High-resolution SEM images along with STEM/EDX of a dolomite-derived sorbent: **a, d** calcined and prior to cyclic testing, **b** 10 cycles of carbonation and calcination, and **c, e** 30 cycles of carbonation and calcination. Scale bars: 200 nm.

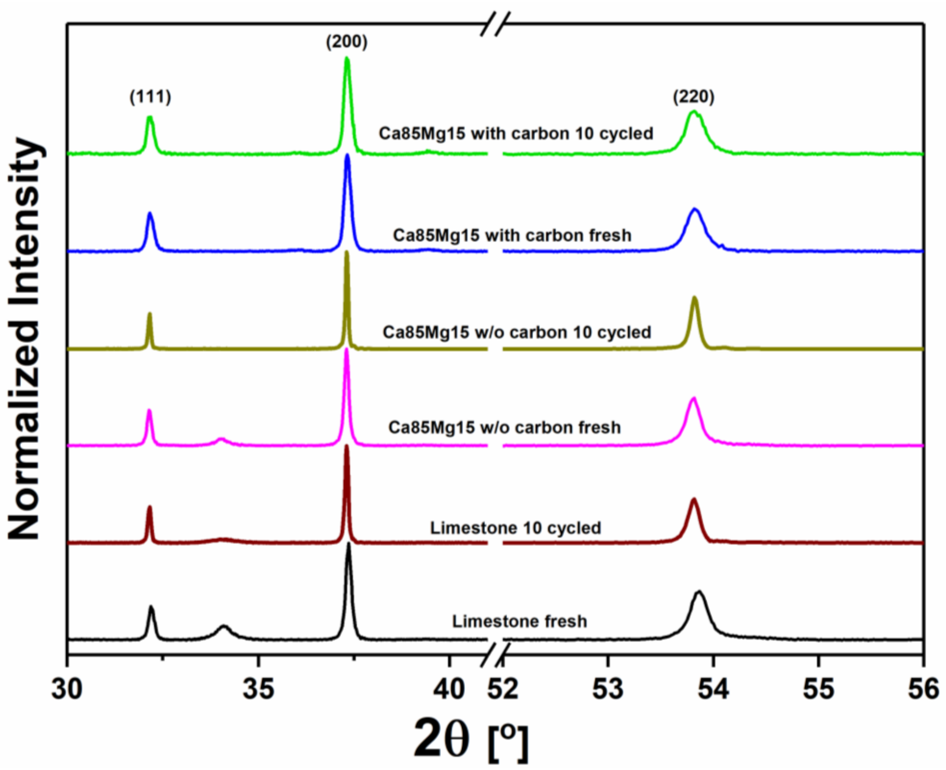

Supplementary Figure 14 Structure-synthesis method relationship. XRD profiles of the hydrothermally synthesized sorbents in the absence and presence of a carbonaceous template compared to limestone-derived CaO (fresh: freshly calcined).

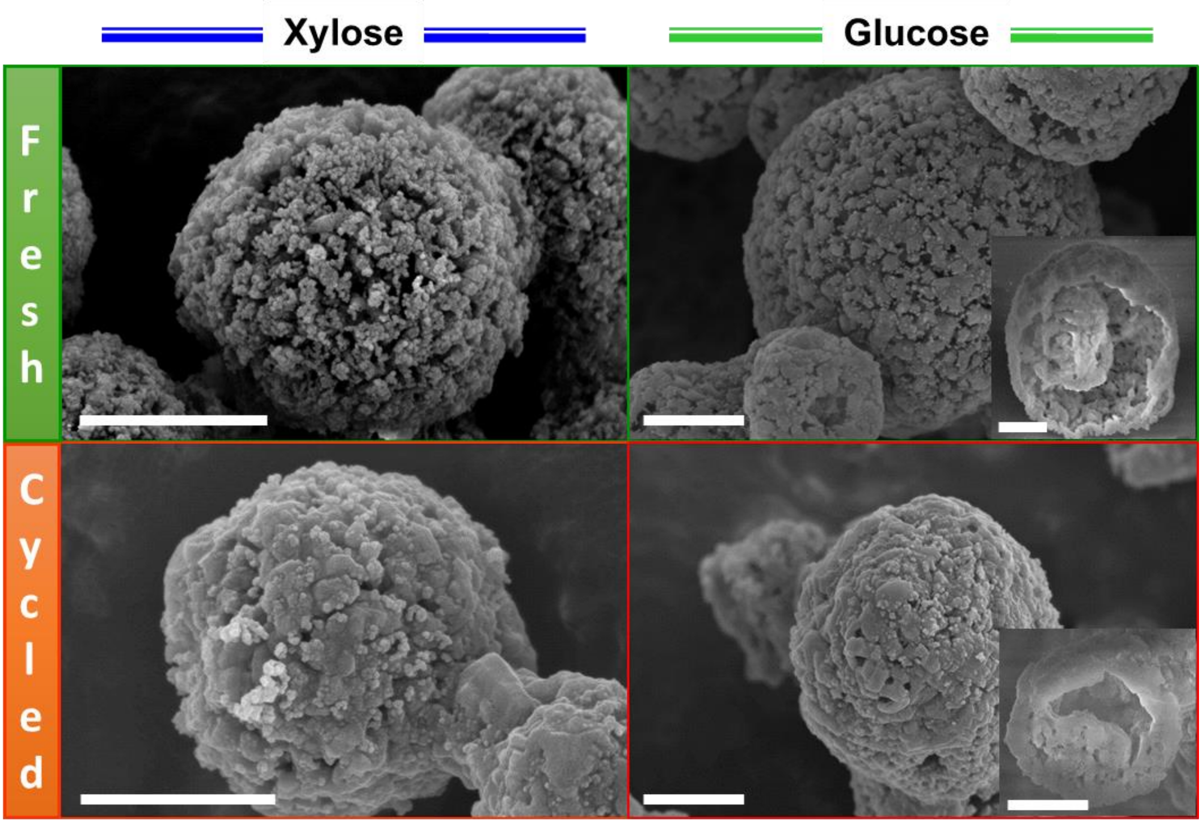

Supplementary Figure 15 Effect of template precursor on the structure of the sorbent. Comparison of the morphology of xylose- and glucose-templated sorbents before (top) and after 10 cycles of carbonation and calcination (bottom). The insets of the glucose-templated sorbent show FIB cross-sections of a microsphere. Scale bars: 1 μm.

Supplementary Table 1 Textural properties of the sorbents. N_2 physisorption of the hydrothermally synthesized sorbents compared to natural limestone and dolomite.

Sorbent	Freshly calcined		10 cycles (calcined state)	
	Surface area[a] (m^2 g^{-1})	Pore volume[b] (cm^3 g^{-1})	Surface area[a] (m^2 g^{-1})	Pore volume[b] (cm^3 g^{-1})
Limestone	15	0.168	2	0.009
Dolomite	18	0.212	7	0.026
Ca100Mg0	8	0.104	2	0.043
Ca90Mg10	13	0.143	5	0.061
Ca85Mg15	17	0.134	7	0.084
Ca85Mg15[c]	22	0.157	2	0.026
Ca80Mg20	27	0.132	8	0.099

[a] Determined by Brunauer-Emmett-Teller (BET) analysis.

[b] Determined by Barrett-Joyner-Halenda (BJH) analysis.

[c] Synthesized hydrothermally in the absence of a carbon precursor.

Supplementary Table 2 Effect of cyclic operation on the crystallite size. Crystallite sizes as determined by XRD using the Scherrer equation.

Sorbent	Crystallite size CaO (200) (nm)	
	Fresh	10 cycled
Limestone-derived CaO	57	> 100
Ca85Mg15 w/o carbon	73	> 100
Ca85Mg15 with carbon	45	48
